# Health economic outcomes and national economic impacts associated with Long COVID in England and Scotland

**DOI:** 10.1007/s10198-025-01788-1

**Published:** 2025-07-09

**Authors:** Joseph Kwon, Joseph Mensah, Ruairidh Milne, Clare Rayner, Román Rocha Lawrence, Johannes De Kock, Manoj Sivan, Stavros Petrou

**Affiliations:** 1 Sheffield Centre for Health and Related Research, University of Sheffield, Sheffield, England United Kingdom; 2 Nuffield Department of Primary Care Health Sciences, University of Oxford, Oxford, England United Kingdom; 3 School of Healthcare Enterprise and Innovation, University of Southampton, Southampton, England United Kingdom; 4 LOCOMOTION Patient Advisory Group Co-Lead, University of Leeds, Sheffield, England United Kingdom; 5 ELAROS 24/7 Ltd, Sheffield, England United Kingdom; 6 COVID Recovery Service, NHS Highland, Inverness, Scotland United Kingdom; 7 Leeds Institute of Rheumatic and Musculoskeletal Medicine, University of Leeds, Leeds, England United Kingdom

**Keywords:** Long COVID, Health utility, Healthcare utilisation, Productivity, Informal caregiving

## Abstract

**Background:**

Two million people in the UK suffer from Long COVID (LC), imposing substantial health economic impacts. This study aimed to: 1) assess longitudinal changes in health utility scores and economic costs of LC, and number of services received at LC specialist clinics and clinic region to capture care intensity; 2) assess whether volume of services received responded to health needs; and 3) estimate the national economic impact of LC.

**Methods:**

LC patients from 10 specialist clinics participated in the LOCOMOTION study. Patient-reported outcomes measures (EQ-5D-5L, C19-YRS and Health Economics Questionnaire) were completed on a digital platform. Associations were assessed between changes in economic outcomes (EQ-5D-3L utility, health economic costs) and number/type of LC specialist services received and region. Per-person values of quality-adjusted life-year losses, public sector costs, productivity losses and informal care costs were multiplied by LC prevalence to estimate national economic impacts.

**Results:**

There was a statistically significant reduction in public sector costs over time. There was no significant association between the number of specialist services received and change in health utility scores. LC specialist clinic and outpatient service utilisation corresponded to health need and had significant regional variation after controlling for health need. LC is associated with a substantial economic impact nationally, estimated at £8.1 billion annually and £24.2 billion since its emergence, comparable to the annual cost of £9.4 billion for stroke.

**Conclusion:**

The effectiveness of LC specialist clinic services warrants further research. The substantial national economic impact of LC warrants a nationwide LC care strategy.

**Supplementary Information:**

The online version contains supplementary material available at 10.1007/s10198-025-01788-1.

## Background

As of March 2024, around two million people in the UK suffer from Long COVID (LC), around 381,000 (19.2%) of whom reported their ability to undertake daily activities to have been limited ‘a lot’ [1]. Across the 38 member countries of the Organisation for Economic Cooperation and Development (OECD), LC prevalence was estimated to be around 39 million (using the most conservative country-specific prevalence estimates), with the UK prevalence rate per 100,000 population being close to the OECD average [2]. Symptoms of LC include fatigue, neurocognitive dysfunction, pain, breathlessness, exercise intolerance, and functional disability [3–6]. The condition can be debilitating, with some patients suffering functional impairments worse than for stroke and comparable to Parkinson’s disease and having lower health-related quality of life (HRQoL) than metastatic cancer [7]. Nearly a third of LC patients reported being unable to live alone without assistance [8]. Seven million quality-adjusted life years (QALYs) are estimated to be lost annually among OECD countries due to LC [2].

Furthermore, there is emerging evidence of the substantial health economic impact of LC in different national settings, including significant burden on the healthcare sector [9–12] and on the wider economy via productivity loss and informal caregiver burden [7, 13–17]. In Canada, LC patients with neurologic or neuropsychiatric symptoms one year after mild COVID-19 infection recorded a significantly higher number of hospital, emergency department and primary care visits than those without symptoms [12]. In the Netherlands, the healthcare utilisation rate was positively associated with the number and severity of LC symptoms [11]. LC patients in Israel incurred 15% higher healthcare costs relative to the pre-infection period [9]. In a multinational cohort, 22% of LC patients had dropped out of the labour market due to symptoms, while 45% had reduced working hours [14]. One US-based modelling study estimated that the rising prevalence of myalgic encephalomyelitis/chronic fatigue syndrome (ME/CFS) due to LC would increase the annual health economic cost (medical expenses and lost income) of ME/CFS in the US from around $36 billion pre-COVID-19 to $149 billion [18]. Such estimates of the population-level impact of LC (see also [13, 15]) help build a coherent case for a national LC care strategy by demonstrating the magnitude of the condition’s health economic impact that could potentially be reduced through interventions [19, 20].

There is still no pharmacological treatment targeting the aetiology of LC, and thus clinical efforts have focused on LC symptom management, including patient self-led, primary care-led, and specialist-led management strategies [2, 21–24]. For LC patients presenting with complex symptoms and functional impairments, multidisciplinary rehabilitation involving respiratory, cardiology and neurology specialists, supported by occupational therapists, physiotherapists, psychologists, and speech and language therapists, among others, represents current best practice [2, 21]. Nevertheless, more research is needed to verify whether such practice meets the health and care needs of LC patients, improves their health and functional outcomes, and reduces costs incurred by the healthcare sector and wider society.

Using data collected from the LOng COvid Multidisciplinary consortium Optimising Treatments and servIces acrOss the NHS (LOCOMOTION) project [25], the current study first aimed to assess the factors associated with longitudinal changes in preference-based health utility scores and economic costs amongst LC patients referred to LC specialist clinics. Here, the key factors to be assessed were the volume and type of services received at LC specialist clinics and whether there was any regional variation in the outcome trajectories across the participating clinics. The related hypotheses were that: greater intensity of care received at LC specialist clinics, measured by the number of service types received, would be associated with more favourable trajectories of health economic outcomes; and there would be regional variation in the trajectories since the LC specialist clinics in each region offer different service models.

The second aim was to assess whether the services received by LC patients – specifically, services received at LC specialist clinics and outpatient specialist services – corresponded to their health needs. The related hypotheses were that: the number of services would be negatively associated with the HRQoL of LC patients; and that there would be regional variation in the utilisation patterns for both groups of services. The final aim was to estimate the national economic impacts of LC by extrapolating from the economic data obtained in LOCOMOTION. Such estimates could serve as inputs into decision-analytic models that evaluate the cost-effectiveness and broader outcomes of interventions targeting individuals with LC [26].

### Methods

The LOCOMOTION study recruited participants referred to 10 LC specialist clinics located in different regions of the UK (Birmingham, Cardiff, Hertfordshire, Highlands, Leeds, Leicester, London, Newcastle, Oxford and Salford) that implemented unique rehabilitation service models. LOCOMOTION involved multi-stakeholder co-design of multidisciplinary intervention models in each specialist clinic to meet the health and care needs of LC patients and improve their health economic outcomes (e.g., preference-based health utility scores, healthcare utilisation, vocational situation) over time [25]. Ethics approval for LOCOMOTION was obtained from the Bradford and Leeds Research Ethics Committee on behalf of Health Research Authority and Health and Care Research Wales on 6th January 2022 (reference: 21/YH/0276) [25]. Participants provided informed consent for research participation, participant-reported data collection, data analysis, and research publication through the ELAROS Digital Patient Reported Outcome Measures (DPTOM) platform (https://www.elaros.com/) when they enrolled into the study and registered on the system [27].

### Target population and data collection

The target population comprised LC patients newly referred to one of the 10 LC specialist clinics participating in LOCOMOTION and subsequently registered on the DPROM system between 1 st June 2021 and 13th September 2023. The DPROM system included several patient-reported outcome measures (PROMs), including three relevant to this study, namely the EQ-5D-5L, a bespoke Health Economics Questionnaire (HEQ) and the COVID-19 Yorkshire Rehabilitation Scale (C19-YRS) [3].

### Outcomes

#### EQ-5D health utility

The primary outcome was preference-based health utility as a measure of HRQoL which is used in health economic evaluations to capture the ‘quality’ component of QALY [28]. The EQ-5D-5L instrument was included in the DPROM platform and covered five dimensions, namely mobility, self-care, usual activities, pain and discomfort, and anxiety and depression, each with five levels of severity [29]. These were used to describe the respondent’s HRQoL ‘today’, i.e., the day of questionnaire response. At the time of analysis (March 2024), no UK-specific EQ-5D-5L preference-based value set was available to convert the EQ-5D-5L dimension responses into health utility scores. The dimension responses were therefore converted into EQ-5D-3L (an earlier version of the EQ-5D with three response levels for each of the five dimensions) utility scores using an established algorithm recommended for health technology assessment purposes by the National Institute of Health and Care Excellence (NICE) [30]. The index was anchored on a scale where 0 = dead and 1 = full health or no problem on all dimensions; scores below zero were available to describe health states deemed worse than death.

#### Service utilisation and costs

Data on service utilisation and economic costs were obtained from the bespoke HEQ on the DPROM system. The HEQ was designed for LOCOMOTION and is reported in full elsewhere [13]. In brief, the HEQ contained questions on the use of following resources for LC symptoms in the month preceding the questionnaire response (henceforth, ‘previous month’): (i) service(s) received at the LC specialist clinic; (ii) outpatient specialist services; (iii) accident and emergency (A&E) attendance and other secondary care utilisation; (iv) hospital inpatient care; (v) community healthcare; (vi) social service utilisation; and (vii) NHS-prescribed medications. Productivity in terms of paid work hours per week and monthly employment income in the period prior to LC incidence (henceforth, ‘pre-LC period’) and in the previous month were measured. In addition, informal care receipt in the previous month was measured in weekly hours.

Resource inputs were valued by assigning unit costs derived from national compendia according to health technology appraisal guidance [31]. The key sources of unit costs were the 2021/22 *National Schedule of NHS Costs* [32] for hospital services, the *British National Formulary* [33] for prescriptions, and the *Unit Costs of Health and Social Care 2022* compendium [34] for community health and social care services. All costs were expressed in pounds (£) valued in 2022 prices. Where appropriate, costs were inflated to 2022 prices using the NHS Hospital and Community Health Services Pay and Prices Inflation Index [34]. Unit costs for health and social care services are shown in Table A1 in the Supplementary Material.

**Table 1:** Sample characteristics

	EQ-5D-5L sample (N = 1,603)	HEQ sample (N = 729)
Mean age (SD)	48.0 (12.8)	48.6 (11.8)
Female N (%)	1,094 (68.3)	518 (71.1)
Ethnicity N (%) *White* *Minority ethnic* *Missing*	1,224 (76.4) 179 (11.2) 200 (12.5)	602 (82.6) 81 (11.1) 46 (6.3)
Region N (%) *Birmingham* *Cardiff* *Hertfordshire* *Highlands* *Imperial* *Leeds* *Leicester* *Newcastle* *Oxford* *Salford*	59 (3.7) 299 (18.7) 67 (4.2) 19 (1.2) 52 (3.2) 571 (35.6) 166 (10.4) 82 (5.1) 96 (6.0) 192 (12.0)	47 (6.5) 71 (9.7) 66 (9.1) 15 (2.1) 39 (5.4) 158 (21.7) 109 (15.0) 63 (8.6) 76 (10.4) 85 (11.7)
IMD quintile N (%) *Most deprived* *2nd* *3rd* *4th* *Least deprived* *Missing*	141 (8.8) 89 (5.6) 119 (7.4) 135 (8.4) 165 (10.3) 954 (59.5)	107 (14.7) 73 (10.0) 95 (13.0) 105 (14.4) 131 (18.0) 218 (29.9)
Mean LC duration in days (SD) [N]	426.1 (277.9) [N = 1,186]	492.5 (287.4) [N = 664]
LC duration N (%) < *1 year* *1–2 years* > *2 years* *Missing*	634 (39.6) 353 (22.0) 199 (12.4) 417 (26.0)	282 (38.7) 236 (32.4) 146 (20.0) 65 (8.9)
Hospitalised for COVID-19 N (%)	147 (9.2)	71 (9.7)
Mean hospitalisation length in days (SD)	12.0 (24.0)	9.3 (14.3)
ICU stay for COVID-19 N (%)	33 (2.1)	16 (2.2)
Mean ICU stay length in days (SD)	14.6 (14.4)	9.3 (13.6)
COVID-19 vaccination N (%) *Double vaccinated* *Single vaccinated* *Missing*	471 (29.4) 78 (4.9) 1,054 (65.8)	395 (54.2) 59 (8.1) 275 (37.7)
Healthcare worker N (%)	228 (14.2)	92 (12.6)
Mean EQ-5D-3L utility (SD) [N]	0.527 (0.265) [N = 1,579]	0.516 (0.278) [N = 622]
Mean C19-YRS functional disability subscale (SD) [N]	7.0 (3.7) [N = 1,100]	7.2 (3.8) [N = 439]
Mean C19-YRS symptom severity subscale (SD) [N]	17.8 (5.8) [N = 1,100]	17.9 (5.9) [N = 439]
Mean C19-YRS overall health subscale (SD) [N]	4.5 (1.9) [N = 1,091]	4.5 (1.9) [N = 438]
Mean C19-YRS other symptoms subscale (SD) [N]	5.2 (4.4) [N = 1,100]	5.8 (4.7) [N = 439]

Abbreviations: C19-YRS: Covid-19 Yorkshire Rehabilitation Scale; HEQ: health economics questionnaire; ICU: intensive care unit; IMD: index of multiple deprivation; SD: standard deviation.

The human capital approach was used to assign monetary values to productivity losses wherein a reduction in each paid work hour was valued at the hourly wage [35]. As done previously [13], for participants that reported their monthly work income and weekly paid work hours in the pre-LC period, this information was used to estimate their pre-infection hourly wage, assuming constant weekly work hours and four working weeks over the income-earning month. This hourly wage was used to calculate the productivity values in both the pre-LC period and for the month preceding the questionnaire response, with the difference between the values being the measure of productivity loss in the previous month relative to the pre-LC period. For informal care, each hour of informal caregiving was valued using the proxy goods method [36]: i.e., it was assumed that in the absence of informal care, individuals would purchase private care as a substitute at the average hourly cost of £20 [37].

For analyses, the costs associated with outpatient specialist settings, other secondary care, inpatient care, community healthcare, social care, NHS-prescribed medications and LC specialist clinic care were combined to generate public sector costs, while economic values associated with productivity losses and informal care were combined to generate wider (i.e., non-public sector) costs. Public sector and wider costs were then combined to inform total societal costs.

### Covariates

#### COVID-19 Yorkshire Rehabilitation Scale (C19-YRS)

The C19-YRS (modified version) is a validated patient-reported outcome measure for LC symptom severity, functional disability and overall health [3]. Its 17 items produced four subscale scores: (i) symptom severity (range 0–30, higher score more severe); (ii) functional disability (0–15, higher score more disabled); (iii) overall health (0–10, higher score better health); and (iv) other symptoms (0–25, higher score more severe). For subscales (i) to (iii), participants were asked to describe their state ‘now’ and their state in the pre-LC period by recall.

#### Demographic, socioeconomic and COVID-19 history variables

Participants reported their demographic and socioeconomic characteristics and COVID-19 history, including: age (integer in years), sex (male, female), ethnicity (White, Asian, Black, mixed, or other), region (Birmingham, Cardiff, Hertfordshire, Highlands, Leeds, Leicester, London, Newcastle, Oxford, or Salford), postcode from which area-based Index of Multiple Deprivation (IMD) decile was derived [38], date(s) of SARS-CoV-2 infection(s), whether a given infection led to LC (i.e., the incident infection, from the date of which the duration of LC was derived), history of hospitalisation(s) due to acute COVID-19 (including history of intensive care unit admission), and COVID-19 vaccination receipt(s) and their date(s) and brand(s). That said, vaccination history was not used as a covariate due to a high rate of missing data.

### Analytic methods

Descriptive statistics of the sample characteristics and the primary outcomes were estimated. Only complete case analyses were conducted given the statistical uncertainty involved in imputing the outcomes and covariates. All statistical analyses were implemented in STATA version 17 [39].

#### Longitudinal analyses

To meet the first study aim, longitudinal changes in the following health economic outcomes were analysed: EQ-5D-3L utility scores computed from EQ-5D-5L dimension responses (a change of 0.03 or above on the utility scale was deemed clinically significant [40]); and health economic costs, categorised as public sector costs, wider costs and total societal costs. Changes were measured between the first and last observations of each respondent. The number of observations and the time duration between the first and last observations differed by respondent. Multiple linear regressions were used to estimate the associations between the changes in economic outcomes and demographic, socioeconomic and COVID-19-related covariates.

The first hypothesis for longitudinal analyses was that greater intensity of care received at LC specialist clinics, measured by the number of service types received, is associated with more favourable trajectories of health economic outcomes: i.e., greater increase in EQ-5D-3L health utility score and greater decline in economic costs. As a corollary, the associations between individual types of services received at LC specialist clinic and the longitudinal trajectories of health economic outcomes were also tested. The second hypothesis was that there would be regional variation in the trajectories across the LC specialist clinics that offer different service models.

The EQ-5D-5L and HEQ PROMs were analysed together to allow: (i) the number of service types received at LC specialist clinic to serve as a covariate for the longitudinal change in EQ-5D-3L utility score; and (ii) the latter to serve as a covariate for the longitudinal changes in economic costs. For (i), the earliest HEQ response taken prior to the last EQ-5D-5L observation was used as a covariate. For (ii), EQ-5D-5L responses taken closest to the dates of the first and last observations for the HEQ (maximum 60 days) were used to calculate the contemporaneous change in EQ-5D-3L utility score.

It was not possible to analyse the EQ-5D-5L, HEQ and C19-YRS PROMs together since the sample size of participants with complete and contemporaneous responses was deemed too small (n < 100). Nevertheless, as a post-hoc analysis, changes in C19-YRS subscale scores were used as covariates for the change in EQ-5D-3L utility score without any HEQ variables as covariates. C19-YRS responses taken closest to the dates of the first and last observations for the EQ-5D-5L (maximum 60 days) were used to calculate the contemporaneous change.

#### Analyses of service utilisation patterns

To meet the second study aim, all responses to the HEQ (not limited to the first and last observations) were combined to be analysed cross-sectionally. Participants were treated as random effects to account for repeated measurements per participant and the other covariates as fixed effects within mixed-effects negative binomial regression models [41]. The first hypothesis was that the number of services received at LC specialist clinics would be associated with the HRQoL of LC patients measured by the EQ-5D-3L utility score, levels of EQ-5D-5L dimensions and C19-YRS subscale scores. Increased service utilisation for participants reporting worse health status would indicate that the service intensity responds to health need. We also estimated the association between the number of outpatient specialist services received and the same health status measures. The second hypothesis was that there would be regional variation in the utilisation patterns for both groups of services. When PROM datasets were analysed together, the EQ-5D-5L and C19-YRS responses closest and prior to each HEQ response were used. This increased the likelihood that the service provisions were responding to health need and not vice versa.

#### Estimation of national economic impact

To meet the final aim, the mean per-person monetary value of QALY loss, public sector cost and wider cost (i.e., productivity loss and informal care cost) incurred between the start of LC and 7th March 2024 – which was the completion date of the Winter Coronavirus Infection Study conducted by the Office for National Statistics (ONS) [1] – were extrapolated to the national level using the LC prevalence data provided by the ONS. Specifically, as of 7th March 2024, 360,221 individuals aged 18 and over in England and Scotland self-reported having LC symptoms that impacted their daily activities ‘a lot’; these estimates were subsequently broken down into age groups (Wave 4 data reported in tab 8 of Excel) [1]. As done previously [13], it was assumed that the LOCOMOTION sample of LC patients referred to LC specialist clinics was representative of this national sub-population with severe LC-related disability.

To estimate the health economic impacts between the start of LC and the first observation timepoint, the bootstrapped mean values (estimated across 1,000 replications) of EQ-5D-3L utility scores and monthly public sector and wider costs were assumed to be constant during this period. Using the mean duration of this period then allowed the estimation of the mean (per-person) accumulated QALY loss, public sector cost and wider cost by age group. The QALY loss was estimated with reference to the UK general population who were assumed to enjoy higher EQ-5D-3L utility scores according to population norms by 10 year wide age groups [42]. Each QALY loss was valued at £20,000 per QALY, which is the cost-effectiveness threshold recommended by NICE [31].

For the period between the first and last observations, the bootstrapped mean values of the longitudinal changes in the above outcomes were used to calculate the mean accumulated outcomes. For the period between the last observation and 7th March 2024, two scenarios were explored: (1) the estimated longitudinal trajectories between the first and last observations were assumed to be maintained after the last observation; and (2) they were not assumed to be maintained such that the bootstrapped mean values of the outcomes at last observation were held constant until 7th March 2024. Further sensitivity analyses were conducted by using the 95% confidence intervals (CIs) for the bootstrapped means of the longitudinal trajectories. Specifically, the bootstrapped 2.5th and 97.5th percentile values were used to estimate, respectively, the lower and upper bounds of each health economic impact. The mean per-person costs accumulated over the three periods were subsequently multiplied by the ONS prevalence per age group to arrive at the total economic impact for England and Scotland.

### Results

There were 1,960 registrations by LOCOMOTION participants on the DPROM system from 1 st June 2021 to 13th September 2023. Of these, 1,603 (81.8%) participants completed the EQ-5D-5L questionnaire at least once and 729 (37.2%) completed the HEQ at least once. These constituted the two study samples, and Table 1 describes their characteristics.

### EQ-5D health utility

From the EQ-5D-5L sample, 790 (49.3%) participants provided at least two observations from which the longitudinal change in EQ-5D-3L utility could be assessed. The mean duration between the first and last observations was 185.7 days (standard deviation (SD) 123.6). The mean change in EQ-5D-3L utility score between the first and last observations was 0.028 (SD 0.197), the difference between this and zero being statistically significant (one-sample *t*-test; *P* < 0.001), while only approaching clinical significance.

Table 2 shows the estimated associations between the change in EQ-5D-3L utility score and the covariates. According to model (1), which adjusted only for variables available in the EQ-5D-5L PROM, namely the LC specialist clinic region and the demographic, socioeconomic and COVID-19 history variables, there was little evidence of any statistically significant association between the change in EQ-5D-3L utility score and the covariates. Model (2) further adjusted for the number of services received at the LC specialist clinics (available from the HEQ PROM) and showed that neither the number of services nor region had statistically significant association with the change in utility score. Results of models (1) and (2) suggested that the two hypotheses for the longitudinal analysis should be rejected. There was also no significant association between individual types of services received at the LC specialist clinics and the change in utility score (results not shown).

**Table 2:** Associations between longitudinal change in EQ-5D-3L utility score and covariates

Model type: multiple linear regression^a^	Model (1): EQ-5D-5L sample [N = 790]		Model (2): Number of LC specialist services as covariate [N = 494]		Model (3): C19-YRS subscale scores as covariates [N = 574]	
	Coeff. (SE)	*P*-value	Coeff. (SE)	*P*-value	Coeff. (SE)	*P*-value
Constant	0.089* (0.034)	0.010	0.103* (0.048)	0.032	0.064 (0.037)	0.085
Duration between 1 st and last response	< 0.001 (< 0.001)	0.995	< 0.001 (< 0.001)	0.402	< 0.001 (< 0.001)	0.402
Age group (ref.: age < 35 years)						
*35–44 years*	−0.034 (0.024)	0.154	−0.043 (0.032)	0.174	−0.034 (0.025)	0.184
*45–54 years*	−0.022 (0.023)	0.331	−0.044 (0.030)	0.142	−0.042 (0.024)	0.086
*55–64 years*	−0.003 (0.024)	0.913	−0.028 (0.031)	0.368	−0.018 (0.026)	0.491
*65–74 years*	−0.024 (0.036)	0.503	−0.048 (0.044)	0.275	−0.050 (0.038)	0.195
≥ *75 years*	−0.057 (0.066)	0.385	−0.055 (0.101)	0.585	−0.013 (0.063)	0.832
Female (ref.: male)	0.004 (0.015)	0.801	−0.003 (0.020)	0.877	−0.012 (0.017)	0.479
Ethnicity (ref.: white)						
*Minority ethnic*	−0.019 (0.023)	0.417	−0.003 (0.031)	0.913	−0.034 (0.025)	0.186
*Missing data*	−0.029 (0.029)	0.322	−0.058 (0.041)	0.162	−0.086* (0.035)	0.015
IMD quintile (ref.: least deprived)						
*2nd*	−0.042 (0.027)	0.117	−0.004 (0.031)	0.909	−0.020 (0.028)	0.487
*3rd*	−0.041 (0.029)	0.158	−0.012 (0.032)	0.704	−0.036 (0.029)	0.214
*4th*	−0.059 (0.033)	0.073	−0.038 (0.037)	0.309	−0.033 (0.034)	0.338
*Most deprived*	−0.058 (0.029)	0.051	−0.047 (0.034)	0.168	−0.025 (0.031)	0.419
*Missing data*	−0.056* (0.024)	0.021	−0.013 (0.033)	0.696	−0.023 (0.027)	0.387
LC duration (ref.: < 1 year)						
*1–2 years*	0.001 (0.019)	0.959	0.008 (0.023)	0.745	0.004 (0.021)	0.830
> *2 years*	0.027 (0.023)	0.232	0.050 (0.027)	0.066	0.031 (0.024)	0.191
*Missing data*	0.023 (0.023)	0.325	0.012 (0.030)	0.690	0.058* (0.027)	0.033
Region (ref.: Leeds)						
*Birmingham*	0.032 (0.036)	0.381	0.026 (0.041)	0.535	−0.032 (0.052)	0.532
*Cardiff*	−0.020 (0.027)	0.458	−0.037 (0.039)	0.337	0.023 (0.036)	0.530
*Hertfordshire*	−0.033 (0.032)	0.300	−0.030 (0.036)	0.408	−0.032 (0.033)	0.342
*Highlands*	0.017 (0.069)	0.800	0.001 (0.080)	0.985	0.053 (0.065)	0.414
*Imperial*	0.025 (0.039)	0.520	0.003 (0.047)	0.949	0.046 (0.039)	0.231
*Leicester*	−0.048 (0.024)	0.050	−0.038 (0.031)	0.215	−0.027 (0.030)	0.382
*Newcastle*	0.028 (0.035)	0.436	0.006 (0.042)	0.884	0.032 (0.039)	0.409
*Oxford*	0.027 (0.028)	0.339	0.034 (0.033)	0.311	0.053 (0.033)	0.102
*Salford*	−0.015 (0.030)	0.601	−0.022 (0.038)	0.559	0.003 (0.041)	0.940
Hospitalised for COVID-19 (ref: not hospitalised)	−0.012 (0.024)	0.612	−0.004 (0.030)	0.900	−0.016 (0.027)	0.561
Number of LC specialist services received			−0.002 (0.007)	0.737		
Change in C19-YRS functional disability subscale^b^					−0.017** (0.004)	< 0.001
Change in C19-YRS symptom severity subscale^b^					−0.008** (0.003)	< 0.001
Change in C19-YRS general health subscale^c^					0.012* (0.005)	0.022
Change in C19-YRS other symptoms subscale^b^					−0.003 (0.003)	0.307

^a^Statistical significance: * *P* < 0.05; ** *P* < 0.01

^b^Higher subscale score implies worse functional disability or greater severity of symptoms

^c^Higher subscale score implies better general health

Abbreviations: C19-YRS: Covid-19 Yorkshire Rehabilitation Scale; coeff.: coefficient; IMD: index of multiple deprivation; LC: Long COVID; ref.: reference; SE: standard error.

As post-hoc analysis, model (3) found that the change in utility score was significantly associated in expected directions with changes in symptom severity, functional disability, and overall health subscale scores for the C19-YRS.

### Economic costs

From the HEQ sample, 269 (36.9%) participants provided at least two observations with complete economic cost data from which the longitudinal change in costs was assessed. The mean duration between the first and last observations was 118.3 days (SD 73.8). The monthly total economic cost declined from a mean of £1,706.6 at the first observation to a mean of £1,416.4 at the last observation, and this mean decline of £290.2 (SD 2,229.6) was significantly different from zero (one-sample *t*-test; *P* = 0.034).

**Fig. 1:**
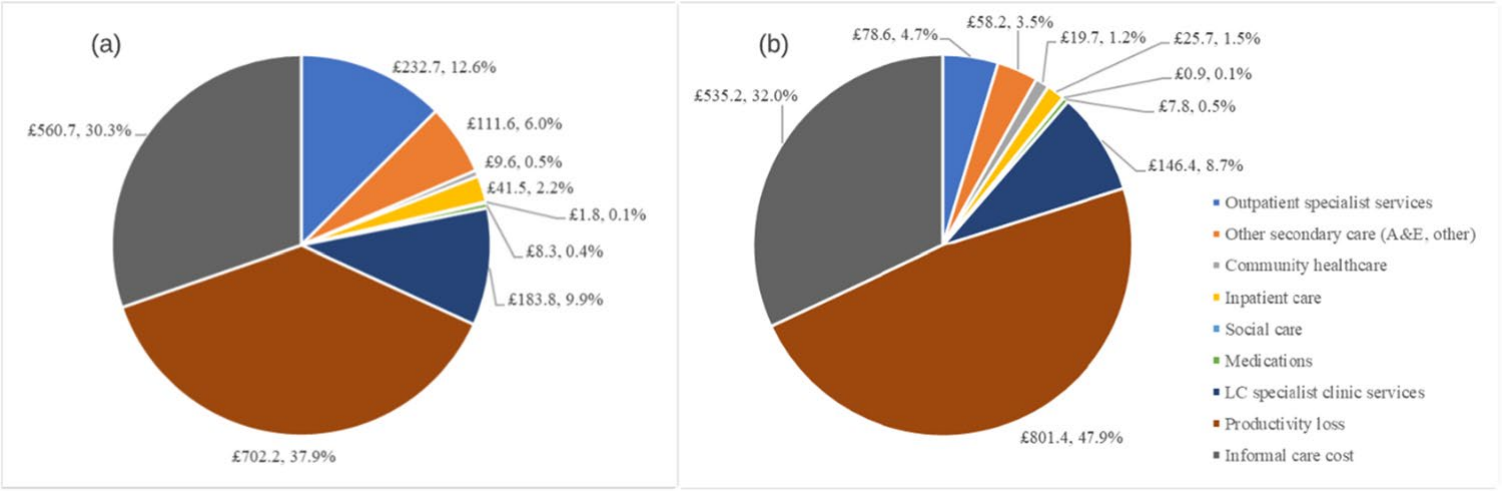
Monthly economic costs at: (a) first observation; (b) last observation. **Abbreviation:** A&E: accident and emergency; LC: Long COVID

Figure 1 breaks down the monthly costs at (a) first and (b) last observations by cost type. At both observations, the monetary value of productivity loss comprised the greatest proportions of the costs (37.9% at first, 47.9% at last), followed by informal care cost (30.3% at first, 32.0% at last). All public sector costs except community healthcare costs saw absolute declines in their monthly mean values between the two observations. There was an absolute increase in the economic value of productivity loss and a slight absolute decrease in informal care cost; they both comprised greater relative proportions at the last observation.

Table 3 shows the estimated associations between changes in the three types of economic costs and covariates. Additional number of services received at LC specialist clinic as reported at first observation was significantly associated with a greater decline in public sector costs in model (1) but not with wider costs in model (2). The negative and statistically significant association with the number of services remained for total societal costs in model (3). Hence, evidence for the first hypothesis was mixed. Contrary to the second hypothesis, there was no evidence of significant association between longitudinal changes in economic costs and region.

**Table 3:** Associations between longitudinal changes in health economic costs and covariates

Model type: multiple linear regression^a^	Model (1): public sector cost [N = 219]		Model (2): wider cost^b^ [N = 219]		Model (3): total societal cost^c^ [N = 219]	
	Coeff. (£) (SE)	*P*-value	Coeff. (£) (SE)	*P*-value	Coeff. (£) (SE)	*P*-value
Constant	146.1 (549.3)	0.791	345.6 (594.0)	0.561	524.3 (865.6)	0.545
Duration between 1 st and last response	0.2 (1.2)	0.883	0.1 (1.3)	0.920	−0.4 (1.9)	0.819
Age group (ref.: age < 35 years)						
*35–44 years*	335.5 (369.1)	0.365	226.0 (399.2)	0.572	451.9 (581.7)	0.438
*45–54 years*	323.1 (344.6)	0.350	−153.2 (372.7)	0.681	133.7 (543.1)	0.806
*55–64 years*	224.3 (360.9)	0.535	−231.8 (390.3)	0.553	−80.4 (568.8)	0.888
*65–74 years*	−226.5 (464.0)	0.626	−239.2 (501.8)	0.634	−574.8 (731.2)	0.433
≥ *75 years*	149.0 (984.4)	0.880	−1,665.6 (1,064.5)	0.119	−1,884.1 (1,551.2)	0.226
Female (ref.: male)	214.6 (191.1)	0.263	−358.7 (206.6)	0.084	−71.4 (301.1)	0.813
Ethnicity (ref.: white)						
*Minority ethnic*	−86.1 (290.8)	0.767	−332.2 (314.5)	0.292	−387.3 (458.2)	0.399
*Missing data*	25.2 (347.6)	0.942	479.4 (375.9)	0.204	410.7 (547.8)	0.454
IMD quintile (ref.: least deprived)						
*2nd*	−200.7 (294.7)	0.497	−270.4 (318.7)	0.397	−504.1 (464.4)	0.279
*3rd*	−354.0 (349.5)	0.312	−559.0 (377.9)	0.141	−907.2 (550.7)	0.101
*4th*	−61.3 (350.6)	0.861	−649.8 (379.2)	0.088	−793.0 (552.5)	0.153
*Most deprived*	−413.6 (325.3)	0.205	−470.3 (351.7)	0.183	−1,094.4* (512.5)	0.034
*Missing data*	−310.5 (332.5)	0.352	−159.9 (359.6)	0.657	−559.7 (524.0)	0.287
LC duration (ref.: < 1 year)						
*1–2 years*	−196.6 (218.1)	0.369	288.8 (235.9)	0.222	67.5 (343.8)	0.845
> *2 years*	91.3 (281.5)	0.746	335.9 (304.4)	0.271	372.4 (443.5)	0.402
*Missing data*	−425.7 (322.6)	0.189	124.3 (348.8)	0.722	−274.6 (508.3)	0.590
Region (ref.: Leeds)						
*Birmingham*	14.7 (395.8)	0.970	536.1 (428.0)	0.212	566.1 (623.8)	0.365
*Cardiff*	−415.9 (413.2)	0.315	−165.9 (446.8)	0.711	−452.5 (651.1)	0.488
*Hertfordshire*	−234.5 (363.2)	0.519	829.4* (392.7)	0.036	612.7 (572.3)	0.286
*Highlands*	No data	N/A	No data	N/A	No data	N/A
*Imperial*	449.8 (467.6)	0.337	109.0 (505.6)	0.830	719.0 (736.8)	0.330
*Leicester*	−57.9 (313.2)	0.854	601.5 (338.7)	0.077	743.6 (493.6)	0.134
*Newcastle*	−789.1 (416.9)	0.060	626.4 (450.8)	0.166	47.5 (656.9)	0.942
*Oxford*	−323.0 (335.0)	0.336	199.3 (362.3)	0.583	−68.9 (527.9)	0.896
*Salford*	81.2 (346.8)	0.815	−212.0 (375.0)	0.572	59.6 (546.4)	0.827
Hospitalised for COVID-19 (ref: not hospitalised)	−128.7 (289.8)	0.658	−161.0 (313.3)	0.608	−311.8 (456.6)	0.496
Number of LC specialist services received at first observation	−203.3** (62.5)	0.001	−74.7 (67.6)	0.270	−279.8** (98.5)	0.005
Change in EQ-5D-3L utility score^d^	2.7 (4.8)	0.575	−5.5 (5.2)	0.295	−6.2 (7.6)	0.421

^a^Statistical significance: * *P* < 0.05; ** *P* < 0.01

^b^Includes monetary values of productivity loss and informal care cost

^c^Includes public sector cost and wider cost

^d^This was calculated by taking the difference between EQ-5D-3L utility scores taken within 60-day intervals around each of the first and last HEQ observations

Abbreviations: coeff.: coefficient; HEQ: health economics questionnaire; IMD: index of multiple deprivation; LC: Long COVID; N/A: not applicable; ref.: reference; SE: standard error.

The lack of association between EQ-5D-3L utility score and the longitudinal changes in economic costs was noteworthy, suggesting that the decline in economic costs, particularly public sector costs, had occurred independently of the change in LC patients’ health status as measured by the EQ-5D-3L. This also meant that the association between the number of services received at LC specialist clinics and public sector costs was present independently of the change in HRQoL.

Table A2 in the Supplementary Material shows the coefficients for the individual types of services received at LC specialist clinics adjusted for the same set of covariates as in Table 3. There were noticeable variations in the association by service type and outcome. For instance, receipt of medical doctor consultations, speech and language therapy, welfare advice, multidisciplinary intervention and sleep management, but not other service types, were significantly associated with declines in public sector costs, while no service type was significantly associated with a decline in wider costs.

### Service utilisation

**Fig. 2:**
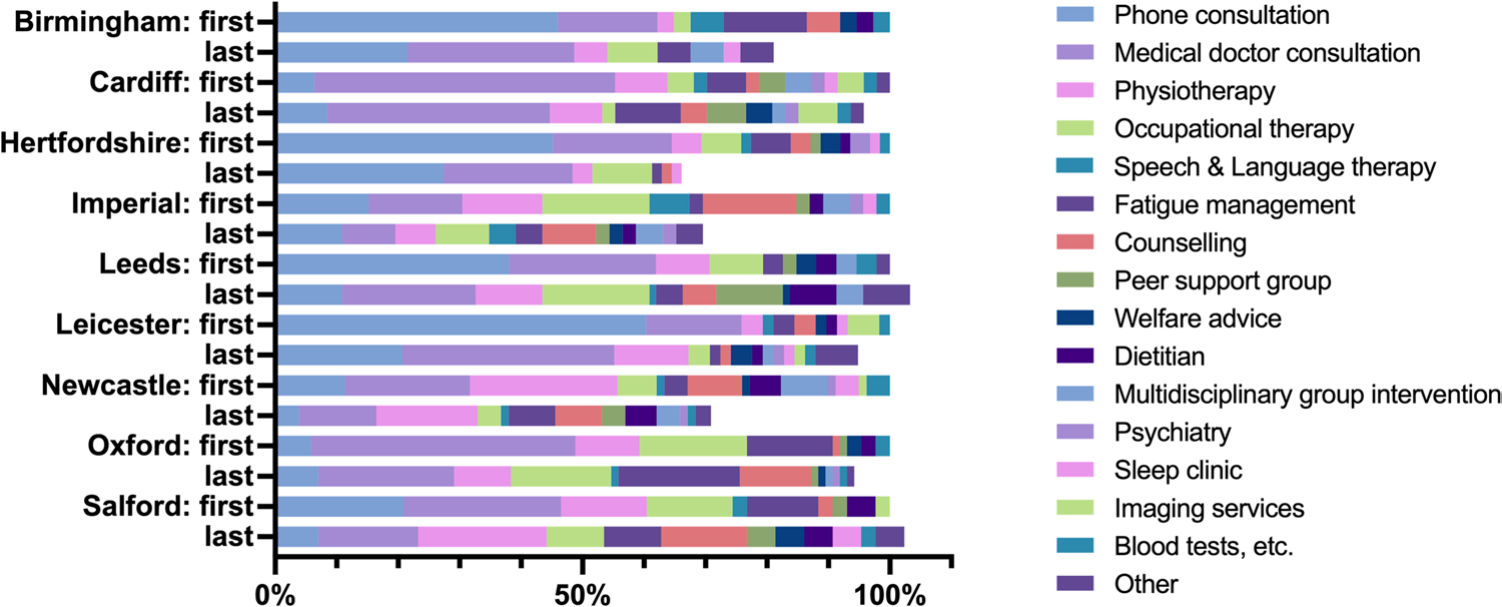
Services received at Long COVID specialist clinics at first and last observations by region. **Note:** the volume of services received at first observation is set at 100% for each region; the Highlands region is excluded because no data was available for the last observation

**Fig. 3:**
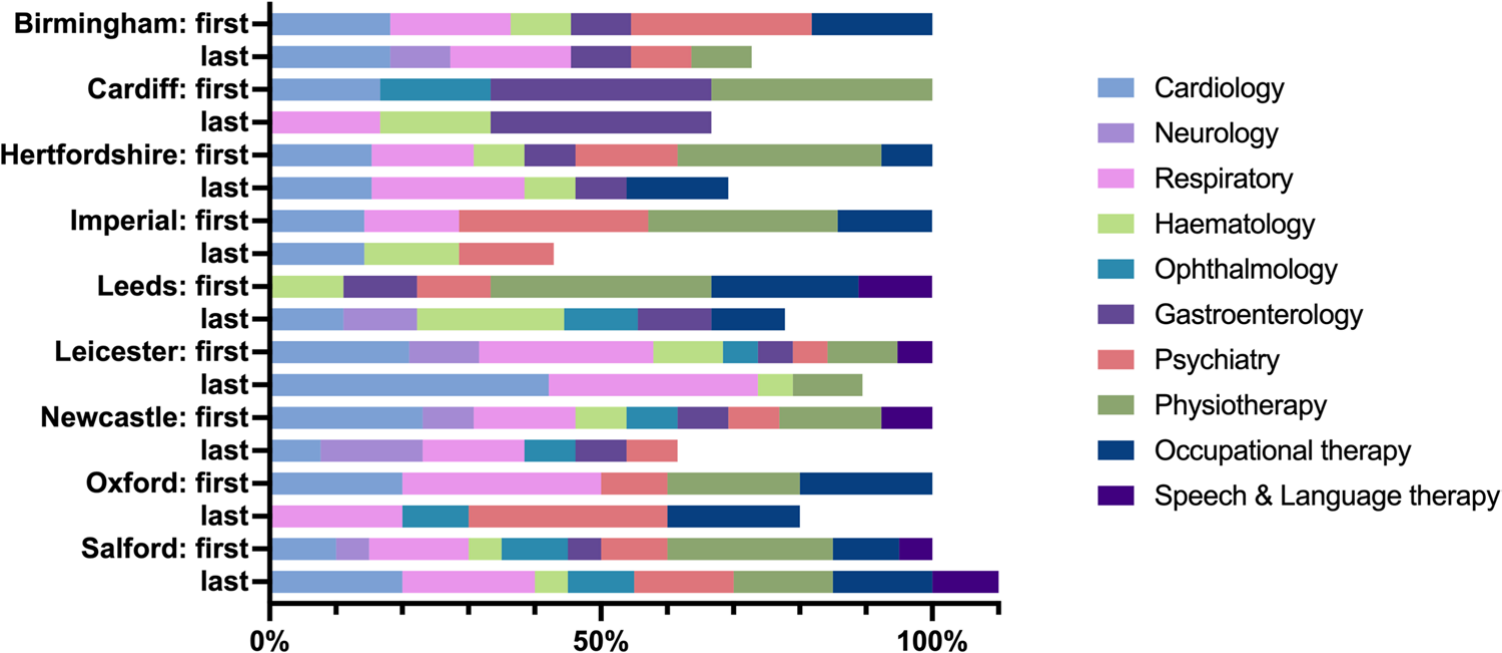
Outpatient specialist services received at first and last observations by region. **Note:** the volume of services received at first observation is set at 100% for each region; the Highlands region is excluded because no data was available for the last observation

Figure 2 shows the range of services received at the 10 LC specialist clinics in the first and last responses to the HEQ, while Fig. 3 shows the range of outpatient specialist services received across the same responses. Few regions saw increases in the volume of service receipt, which explained the decline in public sector costs shown in Fig. 1.

Table 4 shows the results of mixed effects negative binomial regressions testing the hypothesis that the intensity of care received at LC specialist clinics responded to the health needs of LC patients. Adjusted for other covariates, a higher EQ-5D-3L utility score was significantly associated with a lower incidence rate of service receipt at LC specialist clinics. Regarding individual dimensions of the EQ-5D-5L, increased severity of anxiety/depression and pain were significantly associated with higher rates of service receipt after adjusting for other dimensions in model (2). By contrast, none of the C19-YRS subscales was significantly associated with the rate of service receipt when mutually adjusted for each other in model (3).

**Table 4:** Associations between number of services received at LC specialist clinics and covariates

Model type: mixed effects negative binomial regression^a^	Model (1): EQ-5D-3L^b^ index as covariate [N = 1,529]		Model (2): EQ-5D-5L^b^ dimensions as covariates [N = 1,540]		Model (3): C19-YRS^b^ subscales as covariates [N = 1,239]	
	IRR (SE)	*P*-value	Coeff. (SE)	*P*-value	Coeff. (SE)	*P*-value
Constant	2.126** (0.332)	< 0.001	1.085 (0.181)	0.626	1.169 (0.254)	0.472
HEQ response sequence^c^	0.866** (0.014)	< 0.001	0.866** (0.014)	< 0.001	0.860** (0.016)	< 0.001
Age group (ref.: age < 35 years)						
*35–44 years*	1.021 (0.109)	0.842	1.035 (0.110)	0.744	1.024 (0.120)	0.839
*45–54 years*	0.987 (0.099)	0.899	0.997 (0.100)	0.975	0.993 (0.110)	0.952
*55–64 years*	0.961 (0.099)	0.698	0.973 (0.100)	0.789	0.935 (0.107)	0.557
*65–74 years*	0.985 (0.140)	0.914	0.993 (0.140)	0.962	1.104 (0.173)	0.526
≥ *75 years*	1.062 (0.325)	0.843	1.162 (0.284)	0.538	1.462 (0.388)	0.153
Female (ref.: male)	0.947 (0.059)	0.380	0.951 (0.059)	0.418	0.957 (0.068)	0.532
Ethnicity (ref.: white)						
*Minority ethnic*	0.974 (0.096)	0.791	0.967 (0.095)	0.731	0.960 (0.108)	0.718
*Missing data*	1.149 (0.145)	0.271	1.141 (0.141)	0.285	1.237 (0.178)	0.139
IMD quintile (ref.: least deprived)						
*2nd*	1.035 (0.103)	0.730	1.027 (0.100)	0.782	1.101 (0.119)	0.371
*3rd*	1.107 (0.115)	0.329	1.104 (0.114)	0.338	1.166 (0.130)	0.171
*4th*	0.937 (0.110)	0.580	0.930 (0.108)	0.534	1.007 (0.129)	0.957
*Most deprived*	0.966 (0.107)	0.759	0.953 (0.105)	0.660	0.983 (0.119)	0.887
*Missing data*	0.932 (0.102)	0.521	0.918 (0.100)	0.431	0.993 (0.135)	0.961
LC duration (ref.: < 1 year)						
*1–2 years*	1.019 (0.064)	0.766	1.024 (0.064)	0.705	0.987 (0.070)	0.857
> *2 years*	1.006 (0.080)	0.939	1.013 (0.080)	0.871	0.955 (0.084)	0.605
*Missing data*	0.873 (0.108)	0.275	0.872 (0.108)	0.266	0.971 (0.163)	0.862
Region (ref.: Leeds)						
*Birmingham*	1.271 (0.163)	0.062	1.280 (0.163)	0.053	1.236 (0.187)	0.162
*Cardiff*	1.034 (0.143)	0.810	1.044 (0.144)	0.755	0.952 (0.159)	0.770
*Hertfordshire*	1.205 (0.139)	0.106	1.202 (0.137)	0.108	1.283* (0.159)	0.045
*Highlands*	1.189 (0.309)	0.505	1.182 (0.307)	0.519	1.024 (0.303)	0.937
*Imperial*	1.521** (0.208)	0.002	1.522** (0.207)	0.002	1.551** (0.214)	0.001
*Leicester*	0.843 (0.089)	0.108	0.853 (0.090)	0.131	0.886 (0.104)	0.303
*Newcastle*	1.676** (0.187)	< 0.001	1.664** (0.185)	< 0.001	1.540** (0.189)	< 0.001
*Oxford*	1.326** (0.142)	0.008	1.342** (0.144)	0.006	1.258 (0.151)	0.056
*Salford*	0.948 (0.112)	0.650	0.962 (0.113)	0.744	0.942 (0.126)	0.654
Hospitalised for COVID-19 (ref: not hospitalised)	0.844 (0.083)	0.087	0.847 (0.083)	0.090	0.934 (0.101)	0.535
EQ-5D-3L index, multiplied by 100	0.995** (0.001)	< 0.001				
EQ-5D-5L mobility			1.019 (0.041)	0.632		
EQ-5D-5L anxiety			1.071* (0.030)	0.015		
EQ-5D-5L pain			1.074* (0.038)	0.042		
EQ-5D-5L self-care			1.016 (0.041)	0.687		
EQ-5D-5L usual activities			0.999 (0.035)	0.971		
C19-YRS functional disability					1.026 (0.014)	0.057
C19-YRS symptom severity					1.013 (0.009)	0.155
C19-YRS general health					0.987 (0.019)	0.507
C19-YRS other symptoms					1.002 (0.009)	0.848

^a^Statistical significance: * *P* < 0.05; ** *P* < 0.01. Individuals are treated as random effects to account for repeated measurements per individual

^b^Each HEQ response was matched with EQ-5D-5L and C19-YRS responses recorded closest and *prior* to the HEQ response. This increases the likelihood that service receipt is responding to health status and not vice versa

^c^First HEQ response has integer 1, second response 2, and so forth until the *n*^th^ response where *n* varies by individual

Abbreviations: C19-YRS: Covid-19 Yorkshire Rehabilitation Scale; coeff.: coefficient; HEQ: health economics questionnaire; IMD: index of multiple deprivation; IRR: incident rate ratio; LC: Long COVID; ref.: reference; SE: standard error.

That said, when each subscale was included as a separate covariate, the incidence rate ratios (IRRs) were as follows (full results not shown): functional disability score IRR 1.045 (standard error (SE) 0.009; *P* < 0.001); symptom severity score IRR 1.028 (SE 0.006; *P* < 0.001); general health score IRR 0.942 (SE 0.015; *P* < 0.001); and other symptoms score IRR 1.026 (SE 0.007; *P* < 0.001). That is, the rate of service receipt responded to severity of health need as measured by each C19-YRS subscale. The corresponding IRRs for individual dimensions of the EQ-5D-5L were: mobility IRR 1.096 (SE 0.030; *P* = 0.001); anxiety and depression IRR 1.109 (SE 0.028; *P* < 0.001); pain and discomfort IRR 1.123 (SE 0.031; *P* < 0.001); self-care IRR 1.104 (SE 0.034; *P* = 0.001); and usual activities IRR 1.080 (SE 0.028; *P* = 0.003).

Furthermore, there was evidence of significant variation in service receipt rate across regions, with Imperial and Newcastle consistently showing significantly higher rates of service receipt – after controlling for health status – than Leeds across all models.

Another important finding was the consistently significant association between the rate of service receipt and the HEQ response sequence. The latter variable assigned integer 1 to the first HEQ response, integer 2 to the second response, and so forth until the *n*^th^ response where *n* varied by individual. An IRR below 1 for this variable suggested that on average, passage of time – after controlling for health status and other covariates – was associated with a declining rate of service receipt.

Table 5 shows the results of mixed effects negative binomial regressions testing the hypothesis that the intensity of outpatient specialist care responded to the health needs of LC patients. In model (1), a higher EQ-5D-3L utility score was significantly associated with a lower incidence rate of outpatient specialist service receipt. Responses to individual dimensions for the EQ-5D-5L were not significantly associated with the rate of service receipt in model (2), but assessing the dimension responses separately generated the following IRRs: mobility IRR 1.493 (SE 0.133; *P* < 0.001); anxiety and depression IRR 1.261 (SE 0.107; *P* = 0.006); pain and discomfort IRR 1.481 (SE 0.133; *P* < 0.001); self-care IRR 1.469 (SE 0.150; *P* < 0.001); and usual activities IRR 1.456 (SE 0.127; *P* < 0.001). When mutually adjusted in model (3), only the symptom severity subscale of the C19-YRS was significantly associated with the rate of service receipt. When individually assessed, the IRRs were: functional disability IRR 1.110 (SE 0.031; *P* < 0.001); symptom severity IRR 1.088 (SE 0.019; *P* < 0.001); general health IRR 0.914 (SE 0.047; *P* = 0.080); and other symptoms IRR 1.106 (SE 0.026; *P* < 0.001).

**Table 5:** Associations between number of outpatient specialist services received and covariates

Model type: mixed effects negative binomial regression^a^	Model (1): EQ-5D-3L^b^ index as covariate [N = 1,532]		Model (2): EQ-5D-5L^b^ dimensions as covariates [N = 1,540]		Model (3): C19-YRS^b^ subscales as covariates [N = 1,239]	
	IRR (SE)	*P*-value	Coeff. (SE)	*P*-value	Coeff. (SE)	*P*-value
Constant	0.263* (0.146)	0.016	0.027** (0.017)	< 0.001	0.030** (0.022)	< 0.001
HEQ response sequence^c^	0.603** (0.034)	< 0.001	0.601** (0.034)	< 0.001	0.639** (0.039)	< 0.001
Age group (ref.: age < 35 years)						
*35–44 years*	1.053 (0.389)	0.889	1.031 (0.380)	0.934	1.027 (0.412)	0.948
*45–54 years*	1.143 (0.400)	0.703	1.136 (0.395)	0.714	1.201 (0.452)	0.626
*55–64 years*	1.688 (0.599)	0.140	1.646 (0.583)	0.160	1.597 (0.616)	0.225
*65–74 years*	1.360 (0.646)	0.518	1.425 (0.675)	0.454	0.836 (0.454)	0.742
≥ *75 years*	4.851 (4.717)	0.104	3.378 (2.639)	0.119	4.194 (3.499)	0.086
Female (ref.: male)	1.290 (0.275)	0.232	1.274 (0.271)	0.254	0.966 (0.226)	0.883
Ethnicity (ref.: white)						
*Minority ethnic*	0.834 (0.277)	0.585	0.862 (0.287)	0.657	1.019 (0.367)	0.959
*Missing data*	2.096 (0.862)	0.072	2.015 (0.809)	0.081	1.471 (0.711)	0.425
IMD quintile (ref.: least deprived)						
*2nd*	1.692 (0.575)	0.122	1.606 (0.534)	0.154	1.517 (0.564)	0.262
*3rd*	1.945 (0.686)	0.059	1.950 (0.681)	0.056	2.323* (0.856)	0.022
*4th*	1.315 (0.521)	0.490	1.269 (0.498)	0.544	1.517 (0.646)	0.327
*Most deprived*	1.030 (0.401)	0.939	0.998 (0.385)	0.996	1.240 (0.503)	0.596
*Missing data*	1.452 (0.527)	0.304	1.434 (0.517)	0.317	1.175 (0.539)	0.725
LC duration (ref.: < 1 year)						
*1–2 years*	1.348 (0.279)	0.149	1.358 (0.278)	0.136	1.058 (0.241)	0.803
> *2 years*	1.339 (0.348)	0.260	1.330 (0.341)	0.266	1.021 (0.284)	0.941
*Missing data*	0.835 (0.322)	0.640	0.852 (0.326)	0.676	0.418 (0.229)	0.111
Region (ref.: Leeds)						
*Birmingham*	4.361** (1.941)	0.001	4.214** (1.862)	0.001	3.945** (2.015)	0.007
*Cardiff*	3.198* (1.463)	0.011	3.027* (1.377)	0.015	3.476* (1.869)	0.021
*Hertfordshire*	4.982** (1.962)	< 0.001	4.841** (1.882)	< 0.001	4.646** (1.927)	< 0.001
*Highlands*	4.125 (3.397)	0.085	3.902 (3.195)	0.096	7.259* (6.415)	0.025
*Imperial*	3.316* (1.608)	0.013	3.265* (1.576)	0.014	3.348* (1.568)	0.010
*Leicester*	2.959** (1.040)	0.002	2.918** (1.014)	0.002	3.126** (1.172)	0.002
*Newcastle*	1.700 (0.725)	0.213	1.704 (0.723)	0.209	1.481 (0.655)	0.374
*Oxford*	2.721* (1.052)	0.010	2.685* (1.034)	0.010	1.976 (0.858)	0.117
*Salford*	2.110 (0.853)	0.065	2.177 (0.876)	0.053	2.747* (1.193)	0.020
Hospitalised for COVID-19 (ref: not hospitalised)	1.653 (0.509)	0.102	1.683 (0.514)	0.088	1.856 (0.622)	0.065
EQ-5D-3L index	0.984** (0.003)	< 0.001				
EQ-5D-5L mobility			1.168 (0.150)	0.225		
EQ-5D-5L anxiety			1.059 (0.097)	0.535		
EQ-5D-5L pain			1.198 (0.135)	0.110		
EQ-5D-5L self-care			1.060 (0.142)	0.665		
EQ-5D-5L usual activities			1.160 (0.132)	0.193		
C19-YRS functional disability					1.015 (0.044)	0.726
C19-YRS symptom severity					1.062* (0.030)	0.036
C19-YRS general health					1.043 (0.064)	0.487
C19-YRS other symptoms					1.055 (0.031)	0.065

^a^Statistical significance: * *P* < 0.05; ** *P* < 0.01. Individuals are treated as random effects to account for repeated measurements per individual

^b^Each HEQ response was matched with EQ-5D-5L and C19-YRS responses recorded closest and *prior* to the HEQ response. This increases the likelihood that service receipt is responding to health status and not vice versa

^c^First HEQ response has integer 1, second response 2, and so forth until the *n*^th^ response where *n* varies by individual

Abbreviations: C19-YRS: Covid-19 Yorkshire Rehabilitation Scale; coeff.: coefficient; HEQ: health economics questionnaire; IMD: index of multiple deprivation; IRR: incident rate ratio; LC: Long COVID; ref.: reference; SE: standard error.

The regional variations were even more noticeable for outpatient service receipt than for service receipt at LC specialist clinics. The association between HEQ sequence (i.e., time) and declining rate of service receipt remained significant.

### National economic impact

Table 6 shows the estimated per-person mean economic impact by category – QALY loss, public sector cost and wider cost – and age group from the start of LC to 7th March 2024, as well as the extrapolated national impact by the same categories. For instance, under the scenario of the estimated longitudinal trajectories being maintained after the last observation, the mean per-person value of QALY loss was £18,620 for those aged less than 35, which extrapolated to a valuation of around £1.6 billion across England and Scotland. For all age groups, wider costs comprising values of productivity losses and informal care cost consistently made up the largest proportion of the impact. The total national economic impact amounted to around £24.2 billion under the scenario of maintained trajectory and £23.7 billion under the alternative scenario of non-maintained trajectory. These amount, respectively, to around £8.1 billion and £7.9 billion annually.

**Table 6:** Extrapolated national economic impact using the estimated mean longitudinal outcome trajectories

Age group	Mean per-person impact from LC start to 7th March 2024			National prevalence [1]	National impact from LC start to 7th March 2024			National economic impact across outcomes	Annual national economic impact across outcomes^c^
	Monetary value of QALY loss^a^	Public sector cost	Wider cost^b^		Monetary value of QALY loss^a^	Public sector cost	Wider cost^b^		
**Scenario:** constant impact from LC start to first observation; mean longitudinal trajectories between first and last observation maintained after last observation									
< 35 years	£18,620	£13,690	£39,774	99,902	£1,860,185,111	£1,367,662,842	£3,973,501,219	£7,201,349,172	£2,441,135,312
35–44 years	£21,248	£8,712	£46,113	61,638	£1,309,676,589	£536,981,986	£2,842,333,694	£4,688,992,268	£1,547,522,201
45–54 years	£17,673	£9,323	£34,976	70,117	£1,239,177,460	£653,706,576	£2,452,397,316	£4,345,281,351	£1,448,427,117
55–64 years	£14,931	£16,031	£39,354	69,917	£1,043,955,875	£1,120,817,776	£2,751,526,348	£4,916,299,999	£1,591,035,599
65–74 years	£13,064	£15,523	£31,506	37,042	£483,922,349	£575,009,745	£1,167,057,686	£2,225,989,779	£762,325,267
≥ 75 years	£9,117	£9,347	£21,360	21,605	£196,963,036	£201,947,012	£461,475,364	£860,385,412	£301,889,618
Total national impact across age groups								£24,238,297,982	£8,092,335,114
**Scenario:** constant impact from LC start to first observation; mean longitudinal trajectories between first and last observation not maintained									
< 35 years	£20,478	£15,066	£35,544	99,902	£2,045,815,819	£1,505,104,417	£3,550,881,889	£7,101,802,125	£2,407,390,551
35–44 years	£21,603	£11,096	£40,756	61,638	£1,331,571,473	£683,955,465	£2,512,110,931	£4,527,637,869	£1,494,269,924
45–54 years	£18,613	£11,358	£27,649	70,117	£1,305,114,942	£796,382,879	£1,938,633,497	£4,040,131,318	£1,346,710,439
55–64 years	£16,586	£19,462	£30,006	69,917	£1,159,677,037	£1,360,694,683	£2,097,946,981	£4,618,318,701	£1,494,601,521
65–74 years	£13,415	£15,629	£37,286	37,042	£496,925,179	£578,935,937	£1,381,158,297	£2,457,019,413	£841,445,005
≥ 75 years	£8,604	£9,511	£24,678	21,605	£185,895,398	£205,477,132	£533,171,611	£924,544,142	£324,401,453
Total national impact across age groups								£23,669,453,569	£7,908,818,893

^a^QALY loss monetised by using the cost-effectiveness threshold of £20,000 per QALY gained [31]

^b^Includes monetary values of productivity loss and informal care cost

^c^According to EQ-5D-5L sample, the mean durations in years from LC start to 7th March 2024 by age group were: 2.95 years for age < 35 years; 3.03 years for age 35–44 years; 3.00 years for age 45–54 years; 3.09 years for age 55–64 years; 2.92 years for age 65–74 years; and 2.85 years for age ≥ 75 years. These were used to estimate the annual impacts

**Abbreviations:** HEQ: health economics questionnaire; LC: Long COVID; QALY: quality-adjusted life year.

Tables A3 and A4 in the Supplementary Material show respectively the lower and upper bound estimates of the national economic impact. These were £17.8 billion (£5.9 billion annually) and £37.0 billion (£12.4 billion annually), respectively, under the scenario of trajectory maintenance.

## Discussion

This study found evidence of the multidisciplinary nature of the services required to manage this new condition. There was mixed evidence concerning the key hypotheses outlined in relation to the study aims. First, there was no evidence that higher service intensity at LC specialist clinics as measured by the number of service types received is associated with greater improvement in EQ-5D-3L utility score, but there was evidence that higher intensity is associated with faster decline in public sector costs. Second, the volume of service receipt at LC specialist clinics, as well as the volume of outpatient specialist services, appeared to respond to health need as measured by the EQ-5D-3L utility score in particular, although both volumes appeared to fall over time for a reason unrelated to health need. Third, there was no evidence of regional variation in the longitudinal trajectories of health economic outcomes, but there was evidence of significant regional variations in the volumes of LC clinic services and outpatient specialist services. Finally, LC continued to exert substantial burdens on the national workforce, family caregivers and the healthcare system in the order of several billion pounds annually.

The increase in EQ-5D-3L utility score, although statistically significant, failed to reach the threshold of clinical significance: the average rise of 0.028 fell below the magnitude of 0.03 that is typically deemed clinically significant for evaluative purposes [40]. Thus, the average EQ-5D-3L utility score of 0.559 (SD 0.275) at the final observation for the EQ-5D-5L sample remained below that of 229 out of 271 disease groups as categorised under the International Classification of Diseases 9th revision, including brain cancer, schizophrenia and acute myocardial infarction [43]. An important finding was that the longitudinal trajectory of EQ-5D-3L utility score is significantly associated with functional disability and symptom severity subscale scores for the C19-YRS, which suggested that interventions targeting these LC-specific dimensions are likely to improve EQ-5D utility scores. There was also some indication that the volume of services provided at LC specialist clinics responded to deficits in these subscales (when assessed individually). That said, there was no evidence that this generated observable, clinically significant gain in EQ-5D utility score for the study cohort on average.

In contrast, there was strong evidence that greater intensity of services received at LC specialist clinics was associated with declines in public sector costs. Caution is nevertheless required before interpreting this as evidence of intervention effectiveness. First, the overall lack of noticeable improvement in EQ-5D-3L utility score suggests that the decline in public sector cost was not the result of any significant improvement in the health of LC patients. Second, the mixed-effects negative binomial regressions showed the volumes of both LC specialist clinic services and outpatient specialist services falling over time on average, which cannot be explained by the health needs of LC patients. The decline in public sector cost and service utilisation could thus be interpreted as LC patients increasingly struggling to access the requisite care for their needs. Alternatively, a higher intensity of upfront LC specialist clinic services could reduce the need for wider healthcare consultations while the health status of LC patients remained largely unchanged. Further research is required to evaluate the explanatory factors giving rise to these outcome trends.

The evidence of significant regional variations in the number of LC specialist clinic services and outpatient specialist services, after holding health needs constant, was noteworthy. It suggested that the care experience of LC patients with largely comparable needs differed across regions, though the difference appeared not to have affected the longitudinal trajectories of their health economic outcomes. The general picture was that LC patients in several regions received more LC specialist clinic services and outpatient specialist services relative to patients in Leeds. This could be evidence of over-utilisation of resources – and therefore scope for efficiency savings – in regions outside of Leeds or alternatively evidence that some health needs of LC patients – not captured by the EQ-5D-3L utility score, EQ-5D-5L dimension responses and C19-YRS subscale scores – are being addressed in the non-Leeds regions but not in Leeds. Overall, an in-depth comparison of the service models is required to identify the causal mechanisms behind the regional variations observed.

The substantial national health economic impacts estimated in this study corroborated the findings of a previous study using a smaller sample of LOCOMOTION participants [13]. That study valued the national productivity loss at around £3.3 billion annually and the informal care cost at around £2.6 billion annually, equating to £5.9 billion for wider non-public sector costs. In comparison, this study estimated the national wider costs (comprising values of productivity losses and informal care costs) at around £4.6 billion annually, with lower and upper bounds of £2.9 billion and £7.3 billion respectively. An important contribution of this study was the estimation of monetary values of QALY losses and public sector costs, which lifted the total annual cost to £8.1 billion (£5.9 billion lower bound, £12.4 billion upper bound). Wider non-public sector costs therefore comprised over half (56.3%) of the total societal economic burden of LC, which in turn suggests that vocational rehabilitation and family caregiver support should be seen as the central components of LC care strategies [44]. The absolute increase in the monetary value of productivity loss among the LOCOMOTION participants – even while EQ-5D-3L utility score marginally increased and the public sector cost decreased – was particularly concerning and calls for an enhanced focus on vocational rehabilitation. The substantial non-public sector costs also strongly suggested that economic evaluations of LC care strategies should be conducted from a societal perspective to include these outcomes [45, 46]. The parameter estimates obtained from this study, such as the per-person QALY loss, public sector cost and wider cost, could serve as inputs to model-based economic evaluations of LC care strategies from a societal perspective.

To contextualise the national impact estimates, the annual economic costs – including health and social care costs, informal care cost and monetary values of mortality, morbidity and productivity loss – were £20.7 billion (in 2022 prices, inflated from original 2018 prices using the NHS Pay and Prices Inflation Index [34]) for cancer, £13.9 billion for coronary heart disease, £12.8 billion for dementia, and £9.4 billion for stroke in England [47]. The annual cost estimate of £8.1 billion for LC (without including cases whose daily activities were not impaired ‘a lot’) was thus of similar magnitude to that of stroke.

This study contributes to a growing body of literature that evaluates the outcomes of LC patients receiving rehabilitation for their symptoms. Harenwall and colleagues [48] evaluated a psychology-led interdisciplinary virtual rehabilitation for LC patients (n = 76) and found that it significantly improved the EQ-5D-3L utility score by 0.07 (from 0.55 at baseline to 0.62) at the end of the 7-week intervention. However, participants in this study had LC for an average duration of 5.99 months and are thus likely distinct from LOCOMOTION participants who continued to experience persistent symptoms more than 12 months after the disease incidence. Parker and colleagues [49] evaluated a structured pacing protocol given to a group of LC patients (n = 31) with average symptom duration of 17 months and also found a significant increase in EQ-5D-3L utility score by 0.14 (from 0.49 to 0.63) at intervention completion after six weeks. However, the study’s small sample size and short evaluation period hamper generalisation. Smith and colleagues [50] evaluated a 12-week blended digital and community-based LC rehabilitation programme for LC patients (n = 601with an average LC duration of 9.8 months) and found that the intervention significantly increased the EQ-5D-3L utility score by 0.11, reduced volume of GP and outpatient service utilisation, and cut the number of sick days by the end of the intervention period. These results indicate the potential for health improvement and vocational recovery among persistent LC patients, and, further research should compare the service models and patient mix of the LOCOMOTION study and the study by Smith and colleagues [50]. Nonetheless, a common limitation of these intervention studies, including LOCOMOTION, was the absence of control groups, which severely limited the attribution of causal effects.

This study has several key strengths. First, the sample of LC patients included those hospitalised and non-hospitalised for acute COVID-19, with the former comprising a minority of participants (e.g., 9.2% of the EQ-5D-5L sample). This helped us to evaluate the long-term health economic consequences of COVID-19 cases that had mild or moderate acute episodes and yet still posed substantial risk of persistent symptoms, indeed reflecting the majority of LC cases [12, 51, 52]. The lack of significant association between history of hospitalisation for acute COVID-19 and the longitudinal trajectories and service utilisation patterns suggested that hospitalised and non-hospitalised LC patients face broadly comparable health prospects and care needs. Second, the questionnaire-based collection of EQ-5D-5L, productivity and informal caregiving information enabled the estimation of the economic values associated with these outcomes. As the final results showed, excluding these costs – for instance, by solely analysing the healthcare utilisation data obtained from electronic health records – would have greatly underestimated the total economic impact of LC. That said, the participant-reported healthcare utilisation data in this study should be compared against routinely collected records to validate the questionnaire-based data. Third, the statistical analyses in this study adjusted for multiple sociodemographic, socioeconomic and COVID-19 history variables. This found that the longitudinal trajectories of health economic outcomes and the service utilisation patterns were not significantly associated with variables of equity relevance including socioeconomic deprivation and ethnicity. However, this finding should be treated with caution since there is a risk that marginalised populations are underrepresented in our sample [13].

This study nevertheless had some limitations. First, there was high longitudinal attrition of observations such that only a minority of participants provided sufficient data for longitudinal analyses. Still, given that the associations between outcomes and covariates remain unestablished, only complete case analyses were conducted to avoid the statistical uncertainty resulting from any imputation of missing responses using observed data. Second, the digital data collection from the participants did not pre-specify the follow-up timepoints, resulting in individual-level variation in the time interval between the first and last observations. This likely introduced noise when estimating the longitudinal trajectories of outcomes. Third, although the average time intervals between the first and last observations in this study (mean 185.7 days for EQ-5D-5L and 118.3 days for HEQ) were comparable or longer than those in previous LC intervention studies [48–50], a longer follow-up period would have aided the estimation of longitudinal trends. Fourth, the mode of data collection resulted in three separate PROMs, and the matching of contemporaneous responses from them resulted in further data attrition and precluded some analyses: e.g., C19-YRS subscale scores were not used as covariates to explain the longitudinal trajectories of economic costs since the sample with complete contemporaneous responses contained less than 100 individuals. Fifth, the longitudinal analyses took number of service types received at LC specialist clinics as the sole measure of intervention effect. Whether the received service type matched health needs of clinic patients could be used as an alternative measure in further analyses. Sixth, 2022 price levels were used for unit costs of healthcare resource inputs due to the absence of more recent cost catalogues and NHS inflation data at the time of analysis. Given the high inflation since 2022, the nominal values of the economic impacts would be higher than the estimates provided in this study (e.g., £8.1 billion annual cost of LC). Finally, as was the case in previous LC intervention studies [48–50, 53], the lack of a control group hampered the evaluation of any causal effect.

## Conclusion

This study found small improvements in EQ-5D-3L utility score in LC patients receiving care in LC clinics. There was no significant association between the number of service types received at LC specialist clinics and changes in health status. By contrast, higher number of service types was significantly associated with reduction in public sector costs, though caution is warranted before interpreting this as evidence of intervention effectiveness. The volume of service received at LC specialist clinics and non-LC outpatient specialist settings appeared to respond to health needs of LC patients even though there was significant regional variation after controlling for health need. It was unclear whether this variation represented over- or under-utilisation of care resources in specific regions. Finally, LC imposed a substantial economic burden on the national workforce, family caregivers and the public sector with the monetary value of this combined burden estimated to be around £8.1 billion annually and £24.2 billion in total since the emergence of LC. This calls for concerted efforts to implement nationwide LC care strategies, with particular focus on vocational rehabilitation for these patients.

## Supplementary Information

Below is the link to the electronic supplementary material.

Supplementary file1 (DOCX 62 KB)

## Abbreviations

C19-YRS: Covid-19 Yorkshire Rehabilitation Scale
CI: Confidence interval
DPROM: Digital Patient Reported Outcome Measure
HEQ: Health economics questionnaire
ICU: Intensive care unit
IMD: Index of multiple deprivation
IRR: Incidence rate ratio
LC: Long COVID
NICE: National Institute for Health and Care Excellence
ONS: Office for National Statistics
SD: Standard deviation
SE: Standard error
