# Supplementary material for: Health economic outcomes and national economic impacts associated with Long COVID in England and Scotland
Source: Eur J Health Econ. 2025 Jul 9;27(1):149–66. doi: 10.1007/s10198-025-01788-1 (PMC12929292; doi:10.1007/s10198-025-01788-1)
Supplement: Supplementary file 1 — Supplementary file1 (DOCX 62 KB) [file 10198_2025_1788_MOESM1_ESM.docx]

# Health economic outcomes and national economic impacts associated with Long COVID in England and Scotland

**Supplementary Material**

# Unit costs

| **Table A1** Unit costs used for costing of health and social care utilisation | | | |
| --- | --- | --- | --- |
|  | **Unit cost (£ 2022)^a^** | | **Source^b^** |
| **Resource use** | Face | Remote |  |
| **Secondary care** |  |  |  |
| Accident and Emergency (A&E) | 242 | 242 | NHS reference costs 2021/22: 'Index' |
| Cardiology | 284.41 | 173.51 | NHS reference costs 2021/22: 'OP' |
| Neurology | 409.07 | 271.64 | NHS reference costs 2021/22: 'OP' |
| Respiratory | 264.84 | 198.57 | NHS reference costs 2021/22: 'OP' |
| Haematology | 312.95 | 203.15 | NHS reference costs 2021/22: 'OP' |
| Ophthalmology | 159.5 | 111.78 | NHS reference costs 2021/22: 'OP' |
| Gastroenterology | 264.65 | 246.54 | NHS reference costs 2021/22: 'OP' |
| Psychiatry | 265.24 | 170.48 | NHS reference costs 2021/22: 'OP' |
| Physiotherapy | 105.04 | 82.15 | NHS reference costs 2021/22: 'OP' |
| Occupational therapy | 102.68 | 72.77 | NHS reference costs 2021/22: 'OP' |
| Speech and language therapy | 185.22 | 152.56 | NHS reference costs 2021/22: 'OP' |
| 24hr heart monitor | 61.86 |  | Tests & Investigation Costs 2019/2020 |
| 48hr tape fitted | 61.86 |  | Tests & Investigation Costs 2019/2020 |
| Acupuncture | 50 |  | <http://www.lifemedicineclinic.com/costs.html> |
| Allergy clinic | 324.55 | 156.36 | NHS reference costs 2021/22: 'OP' |
| Anticoagulant | 155.65 | 194.49 | NHS reference costs 2021/22: 'OP' |
| Audiology | 140.63 | 103.84 | NHS reference costs 2021/22: 'OP' |
| Birmingham Healthy Minds | 194.5 | 250.9 | NHS reference costs 2021/22: 'OP' |
| Blood test | 4.7 |  | NHS reference costs 2021/22: 'DAPS' |
| Breast Clinic | 142.37 |  | NHS reference costs 2021/22: 'IMAG' |
| CBT | 35 |  | https://www.nhs.uk/mental-health/talking-therapies-medicine-treatments/talking-therapies-and-counselling/counselling/ |
| CBT: Back on Track mental health | 35 |  | https://www.nhs.uk/mental-health/talking-therapies-medicine-treatments/talking-therapies-and-counselling/counselling/ |
| Counselling: initial assessment | 35 |  | https://www.nhs.uk/mental-health/talking-therapies-medicine-treatments/talking-therapies-and-counselling/counselling/ |
| Counselling: via amica at work UHL | 35 |  | https://www.nhs.uk/mental-health/talking-therapies-medicine-treatments/talking-therapies-and-counselling/counselling/ |
| CT scan | 142.37 |  | NHS reference costs 2021/22: 'IMAG' |
| Dermatology | 163.39 | 114.52 | NHS reference costs 2021/22: 'OP' |
| Diabetes clinic | 182.99 | 123.76 | NHS reference costs 2021/22: 'OP' |
| Dietitian | 141.85 | 122.24 | NHS reference costs 2021/22: 'OP' |
| ECG | 61.86 |  | Tests & Investigation Costs 2019/2020 |
| Endocrinology | 304.12 | 189.97 | NHS reference costs 2021/22: 'OP' |
| ENT | 175.21 | 136.03 | NHS reference costs 2021/22: 'OP' |
| Fatigue | 99.11 |  | NHS reference costs 2021/22: 'CHS' |
| Fatigue clinic | 99.11 |  | NHS reference costs 2021/22: 'CHS' |
| Fatigue management workshop | 35 |  | https://www.nhs.uk/mental-health/talking-therapies-medicine-treatments/talking-therapies-and-counselling/counselling/ |
| General health check | 35 |  | Unit cost unclear; assumed same as GP consultation |
| Gynaecology | 215.19 | 169.85 | NHS reference costs 2021/22: 'OP' |
| Hydrotherapy | 50 |  | <https://www.circlehealthgroup.co.uk/treatments/hydrotherapy> |
| Immunology | 536.89 |  | NHS reference costs 2021/22: 'OP' |
| Infectious disease Consultant | 123.03 | 106.60 | NHS reference costs 2021/22: 'OP' |
| ME/CFS Clinic | 293.56 | 293.56 | NHS reference costs 2021/22: 'REHAB' |
| Mental health | 194.5 | 250.9 | NHS reference costs 2021/22: 'OP' |
| Mental health: Leeds Mental Wellbeing Service | 35 |  | https://www.nhs.uk/mental-health/talking-therapies-medicine-treatments/talking-therapies-and-counselling/counselling/ |
| MRI | 219.04 |  | NHS reference costs 2021/22: 'IMAG' |
| Occupational health | 144 | 38.5 | https://www.hohs.org/costs/ |
| Oncology | 206.47 | 164.22 | NHS reference costs 2021/22: 'OP' |
| Optician | 25 |  | <https://www.specsavers.co.uk/help-and-faqs/how-much-is-an-eye-test> |
| Optometrist | 399.23 | 220.23 | NHS reference costs 2021/22: 'OP' |
| Orthopaedic | 186.01 | 126.51 | NHS reference costs 2021/22: 'OP' |
| Pain management | 293.56 |  | NHS reference costs 2021/22: 'REHAB' |
| Phlebotomy | 4.7 |  | NHS reference costs 2021/22: 'DAPS' |
| Prostrate clinic | 173.66 | 131.69 | NHS reference costs 2021/22: 'OP' |
| Psychology | 392.6 | 383.94 | NHS reference costs 2021/22: 'OP' |
| Pulmonary | 293.56 |  | NHS reference costs 2021/22: 'REHAB' |
| Pulmonary Rehabilitation | 293.56 |  | NHS reference costs 2021/22: 'REHAB' |
| Radiology | 302.95 | 75.96 | NHS reference costs 2021/22: 'OP' |
| Reflexology | 35 |  | https://www.barefoot-reflexology.co.uk/treatments-prices/ |
| Rehabilitation clinic | £293.56 |  | NHS reference costs 2021/22: 'REHAB' |
| Rheumatology | 272.6 | 164.02 | NHS reference costs 2021/22: 'OP' |
| ROH | 35 | 35 | https://www.nhs.uk/mental-health/talking-therapies-medicine-treatments/talking-therapies-and-counselling/counselling/ |
| Silvercloud | 35 |  | https://www.nhs.uk/mental-health/talking-therapies-medicine-treatments/talking-therapies-and-counselling/counselling/ |
| Sleep apnea clinic | 515.98 | 349.65 | NHS reference costs 2021/22: 'OP' |
| Sleep assessment | 184.01 |  | NHS reference costs 2021/22: 'OPROC' |
| Sleep clinic | 515.98 | 349.65 | NHS reference costs 2021/22: 'OP' |
| Sleep support group | 35 | 14.43 | Unit cost unclear; assumed same as GP consultation |
| Sleep sessions | 35 | 14.43 | Unit cost unclear; assumed same as GP consultation |
| Stroke clinic | 631.19 |  | NHS reference costs 2021/22: 'REHAB' |
| Talking therapy: TalkingSpacePlus | 35 |  | https://www.nhs.uk/mental-health/talking-therapies-medicine-treatments/talking-therapies-and-counselling/counselling/ |
| Urogynaecology | 173.66 | 131.69 | NHS reference costs 2021/22: 'OP' |
| Urology | 173.66 | 131.69 | NHS reference costs 2021/22: 'OP' |
| Wound check | 35 | 14.43 | Unit cost unclear; assumed same as GP consultation |
| X-ray | 128.56 |  | NHS reference costs 2021/22: 'IMAG' |
| **Inpatient care** |  |  |  |
| Intensive care unit – short stay | 1753 |  | NHS reference costs 2021/22: 'Index' |
| General ward – short stay | 801 |  | NHS reference costs 2021/22: 'Index' |
| General ward – long stay | 4409 |  | NHS reference costs 2021/22: 'Index' |
| **Community healthcare services** |  |  |  |
| GP appointment | 35 | 14.43 | PSSRU 2022: Table 9.4.2, Table 9.5.1 |
| NHS 111 |  | 14.43 | PSSRU 2022: Table 9.5.1 |
| NHS walk-in centre | 79.79 | 66.93 | NHS reference costs 2021/22: 'EC' |
| Nurse | 11.5 | 2 | PSSRU 2022: Table 9.3.1, Table 9.5.1 |
| District nurse appointment | 53.74 | 39.1 | NHS reference costs 2021/22: 'CHS' |
| Ambulance callout | 276 |  | PSSRU 2022: Table 6.1.1 |
| Mental health nurse appointment | 20 | 20 | PSSRU 2022: Table 2.5.1 |
| Community psychiatrist appointment | 41 | 41 | PSSRU 2022: Table 2.4.1 |
| Health visitor appointment | 11.5 | 2 | PSSRU 2022: Table 9.3.1, Table 9.5.1 |
| Residential care | 198.9 |  | NHS reference costs 2021/22: 'CHS' |
| Asthma nurse | 11.5 |  | PSSRU 2022: Table 9.3.1 |
| Blood pressure check | 11.5 |  | PSSRU 2022: Table 9.3.1 |
| Bowel nurse | 11.5 |  | PSSRU 2022: Table 9.3.1 |
| CAST team | 35 |  | Unit cost unclear; assumed same as GP consultation |
| General health check | 35 |  | Unit cost unclear; assumed same as GP consultation |
| Falls team | 23.5 |  | PSSRU 2022: Table 10.3.1 |
| Health & Wellbeing | 35 |  | https://www.nhs.uk/mental-health/talking-therapies-medicine-treatments/talking-therapies-and-counselling/counselling/ |
| Lung function test | 19.59 |  | NHS reference costs 2021/22: 'DADS' |
| Occupational therapy | 23.5 |  | PSSRU 2022: Table 10.3.1 |
| Pharmacist | 11.5 |  | PSSRU 2022: Table 9.3.1 |
| Psychologist | 41 | 41 | PSSRU 2022: Table 2.4.1 |
| Sleep support group | 35 | 14.43 | Unit cost unclear; assumed same as GP consultation |
| Smoking cessation | 29.31 | 29.31 | PSSRU 2022: Section 7.3 |
| Social worker | 10.5 |  | PSSRU 2022: Table 10.1.1 |
| **Social care services** |  |  |  |
| Care manager appointment | 41 | 41 | PSSRU 2022: Table 10.5.1 |
| Social worker appointment | 42 | 42 | PSSRU 2022: Table 10.1.1 |
| Home care worker appointment | 23 | 23 | PSSRU 2022: Table 10.4.1 |
| Food, laundry and equipment service | 25 |  | PSSRU 2022: Table 10.6.1 |
| Residential care | 206 |  | PSSRU 2022: Table 1.3.1 |
| Disability Team as part of Student Finance England | 35 |  | Unit cost unclear; assumed same as GP consultation |
| Occupational therapy | 23.5 |  | PSSRU 2022: Table 10.3.1 |
| SALFORD Health Improvement | 35 |  | Unit cost unclear; assumed same as GP consultation |
| Assessment for Blue Badge parking | 35 |  | Unit cost unclear; assumed same as GP consultation |
| Weight management |  | 35 | Unit cost unclear; assumed same as GP consultation |
| **LC specialist clinic services** |  |  |  |
| Phone consultation | 36 |  | PSSRU 2022: Table 11.2.2 |
| Medical doctor consultation | 56.5 |  | PSSRU 2022: Table 11.3.2 |
| Physiotherapy | 105.04 |  | NHS reference costs 2021/22: 'OP' |
| Occupational therapy | 102.68 |  | NHS reference costs 2021/22: 'OP' |
| Speech and language therapy | 152.56 |  | NHS reference costs 2021/22: 'OP' |
| Fatigue management | 99.11 |  | NHS reference costs 2021/22: 'CHS' |
| Counselling | 35 |  | https://www.nhs.uk/mental-health/talking-therapies-medicine-treatments/talking-therapies-and-counselling/counselling/ |
| Peer support group | 28.52 |  | NHS reference costs 2021/22: 'CHS' |
| Dietitian | 141.85 |  | NHS reference costs 2021/22: 'OP' |
| Welfare advice | 35 |  | https://www.nhs.uk/mental-health/talking-therapies-medicine-treatments/talking-therapies-and-counselling/counselling/ |
| Multidisciplinary group intervention | 157.98 |  | NHS reference costs 2021/22: 'CHS' |
| Medical doctor consultation | 56.5 | 56.5 | PSSRU 2022: Table 11.3.2 |
| Physiotherapy | 105.4 | 82.15 | NHS reference costs 2021/22: 'OP' |
| Occupational therapy | 102.68 | 72.77 | NHS reference costs 2021/22: 'OP' |
| Speech and language therapy | 152.56 | 185.22 | NHS reference costs 2021/22: 'OP' |
| Fatigue management | 99.11 | 99.11 | NHS reference costs 2021/22: 'CHS' |
| Counselling | 35 | 35 | https://www.nhs.uk/mental-health/talking-therapies-medicine-treatments/talking-therapies-and-counselling/counselling/ |
| Peer support group | 28.52 | 28.52 | NHS reference costs 2021/22: 'CHS' |
| Dietitian | 141.85 | 115.92 | NHS reference costs 2021/22: 'OP' |
| Welfare advice | 35 | 35 | https://www.nhs.uk/mental-health/talking-therapies-medicine-treatments/talking-therapies-and-counselling/counselling/ |
| Multidisciplinary group intervention | 157.98 | 157.98 | NHS reference costs 2021/22: 'CHS' |
| Long COVID nurse | 11.5 |  | PSSRU 2022: Table 9.3.1 |
| Post-COVID clinic online classes | 35 | 35 | Unit cost unclear; assumed same as GP consultation |
| Psychology | 392.6 | 383.94 | NHS reference costs 2021/22: 'OP' |
| Respiratory | 264.84 | 198.57 | NHS reference costs 2021/22: 'OP' |
| Exercise class | 35 | 35 | Unit cost unclear; assumed same as GP consultation |
| Breathing clinic | 35 | 35 | Unit cost unclear; assumed same as GP consultation |
| Long COVID rehabilitation | 293.56 |  | NHS reference costs 2021/22: 'REHAB' |
| **Medications for LC symptoms** | **Unit cost** | **Unit** | **Source**: British National Formulary |
| Allopurinol | 0.03 | 28 | 100mg tablets |
| Amitriptyline hydrochloride | 0.03 | 28 | 10mg tablets (Amitriptyline) |
| Amlodipine | 0.03 | 28 | 5mg tablets |
| Amoxicillin | 0.12 | 15 | 500mg capsules |
| Apixaban | 0.77 | 28 | 5mg tablets |
| Aspirin | 0.03 | 28 | 75mg tablets |
| Atorvastatin | 0.19 | 28 | 80mg tablets |
| Beclometasone dipropionate | 0.04 | 200 | 50 mcg per 1 dose inhaler (Qvar 50) |
| Beclometasone dipropionate | 0.04 | 200 | 100mcg/dose inhaler (Clenil Modulite) |
| Beclometasone with formoterol | 0.24 | 120 | 100mcg/dose inhaler (Fostair Nexthaler) |
| Beclometasone with formoterol | 0.12 | 120 | 100mcg/dose, 6mcg/dose (Luforbec) |
| Benzylpenicillin sodium |  | 2 | 600mg powder (Penicillin) |
| Betahistine dihydrochloride | 0.15 | 84 | 16mg tablets (Betahistine) |
| Betamethasone | 0.22 | 100 | 500mcg tablets (Betnovate) |
| Bisacodyl | 0.08 | 60 | 5mg tablets |
| Bisoprolol fumarate | 0.04 | 28 | 2.5mg tablets (Beta-blockers) |
| Budesonide | 0.07 | 200 | 100 microgram per 1 dose (Turbohaler) |
| Budesonide with formoterol | 0.23 | 120 | 200mcg/dose inhaler (Symbicot) |
| Budesonide with formoterol | 0.18 | 60 | 160mcg/dose, 4.5mcg/dose (Fobumix Easyhaler) |
| Buprenorphine | 4.40 | 4 | 5mcg per hour patch (BuTrans) |
| Buprenorphine | 1.98 | 4 | 5mcg per hour patch (Butec) |
| Buspirone | 0.18 | 30 | 5mg tablets |
| Candersartan cilexetil | 0.04 | 28 | 8mg tablets (Candersartan) |
| Carbocisteine | 0.38 | 60 | 750mg capsules |
| Carbomer | 0.28 | 10 | 0.2% eye gel |
| Cetirizine hydrochloride | 0.03 | 30 | 10mg tablets (Antihistamine) |
| Chloramphenicol | 0.99 | 10 | 5mg per 1 ml |
| Chlorhexidine with neomycin | 0.13 | 15 | 1mg per 1 gram (Naseptin nasal cream) |
| Citalopram | 0.05 | 28 | 20mg tablets |
| Clarithromycin | 0.55 | 14 | 500mg tablets |
| Clopidogrel | 0.05 | 28 | 75mg tablets |
| Co-amoxiclav | 0.33 | 15 | 500mg/125mg tablets (Antibiotics) |
| Co-codamol | 0.03 | 100 | 30mg/500mg tablets (Zapain) |
| Codeine phosphate | 0.04 | 28 | 30mg tablets |
| Colchicine | 0.03 | 100 | 500mcg tablets |
| Colecalciforel | 0.06 | 30 | 800unit capsules |
| Cyanocobalamin | 0.25 | 50 | 50mcg tablets |
| Cyclizine | 0.04 | 100 | 50mg tablets |
| Dapagliflozin | 1.31 | 28 | 5mg tablets |
| Desogestrel |  | 84 | 75mcg tablets |
| Dexamfetamine | 1.33 | 30 | 10mg tablets |
| Diazepam |  | 28 | 2mg tablets |
| Diclofenac potassium | 0.26 | 30 | 50mg tablets (Voltarol) |
| Dihydrocodeine with paracetamol | 0.09 | 30 | 10mg/500mg tablets (Co-dydramol) |
| Diltiazem hydrochloride | 0.13 | 56 | 90mg tablets (Tildiem Retard) |
| Domperidone |  | 30 | 10mg tablets |
| Doxycycline | 0.12 | 8 | 100mg capsules |
| Duloxetine | 0.08 | 28 | 30mg capsules (Anti-depressant) |
| Erythromycin | 0.18 | 100 | 250mg tablets (Erythocin) |
| Esomeprazole | 0.13 | 28 | 20mg tablets |
| Estradiol | 0.08 | 80 | 600mcg per 1 gram gel (Oestrogel) |
| Estradiol | 0.54 | 8 | 75mcg patches (Evorel) |
| Estradiol | 0.14 | 84 | 2mg tablets (Kliofem) |
| Ethinylestradiol with levonorgestrel |  | 63 | (Microgynon) |
| Etoricoxib | 0.09 | 28 | 60mg tablets |
| Famotidine | 0.80 | 28 | 20mg tablets (Pepcid) |
| Felodipine | 0.07 | 28 | 5mg tablets |
| Ferrous fumarate | 0.05 | 84 | 210mg tablets |
| Ferrous sulfate | 0.05 | 28 | 200mg tablets (Iron) |
| Fexofenadine hydrochloride | 0.07 | 30 | 180mg tablets (Fexofenadine) |
| Flecainide acetate | 0.08 | 60 | 100mg tablets (Flecainide) |
| Fluconazole |  | 7 | 50mg capsules |
| Fludrocortisone acetate | 0.24 | 30 | 100mcg tablets (Fludrocortisone) |
| Fluoxetine | 0.04 | 30 | 20mg capsules |
| Fluticasone | 0.05 | 120 | 27.5mcg/dose nasal spray (Avamys) |
| Fluticasone propionate | 0.07 | 150 | 50mcg/dose nasal spray |
| Fluticasone with azelastine | 0.12 | 120 | 137 mcg/dose (Dymista) |
| Fluticasone with salmeterol | 0.17 | 120 | 25mcg/250mcg (AirFluSal) |
| Fluticasone with salmeterol | 0.24 | 120 | 250mcg per 1 dose (Seretide 250 Evohaler) |
| Fluticasone with umeclidinium and vilanterol | 1.48 | 30 | dry powder inhaler (Trelegy Ellipta) |
| Fluticasone with vilanterol | 0.98 | 30 | 184micrograms/dose / 22micrograms/dose dry powder inhaler (Relvar Ellipta) |
| Folic acid | 0.03 | 28 | 5mg tablets |
| Gabapentin | 0.03 | 100 | 300mg capsules |
| Hydroxocobalamin |  | 5 | 1mg/ml (Vitamin B12 injection) |
| Isosorbide mononitrate | 0.10 | 56 | 30mg tablets |
| Ivabradine | 0.56 | 56 | 5mg tablets |
| Lansoprazole | 0.24 | 28 | 30mg tablets |
| Levocarnitine | 0.19 | 60 | 500mg capsules |
| Levothyroxine sodium | 0.03 | 28 | 25mcg tablets (Levothyroxine) |
| Lisdexamfetamine mesilate | 2.45 | 28 | 50mg capsules |
| Loratadine | 0.03 | 30 | 10mg tablets |
| Losartan potassium | 0.03 | 28 | 25mg tablets (Losartan) |
| Mebeverine hydrochloride | 0.07 | 100 | 135mg tablets (Mebeverine) |
| Mefenamic acid |  | 28 | 250mg |
| Melatonin | 0.26 | 30 | 2mg tablets |
| Melatonin | 0.51 | 30 | 2mg tablets (Circadin) |
| Metformin hydrochloride | 0.03 | 28 | 500mg tablets (Metformin) |
| Midodrine hydrochloride | 0.38 | 100 | 5mg tablets (Midodrine) |
| Mirtazapine | 0.04 | 28 | 30mg tablets |
| Mometasone furoate | 0.03 | 140 | 50mcg/dose nasal spray (Mometasone) |
| Montelukast | 0.05 | 28 | 10mg tablets |
| Naproxen | 0.04 | 28 | 250mg tablets |
| Nirmatrelvir with ritonavir | 0.08 | 30 | 150mg/100mg tablets (Paxlovid) |
| Nizatidine | 0.83 | 30 | 150mg capsules |
| Nortriptyline | 0.02 | 100 | 10mg tablets |
| Nutrition supplements | 0.52 | 7 | 57g powder sachets (Foodlink) |
| Nutrition supplements | 2.43 | 4 | 120ml bottle (Altrashot) |
| Nutrition supplements | 1.11 | 5 | 500ml bottle (Altraplen) |
| Olanzapine | 0.03 | 28 | 2.5mg tablets |
| Omeprazole | 0.27 | 28 | 20mg gastro-resistant tablets |
| Ondansetron | 0.95 | 10 | 4mg tablets |
| Pantoprazole | 0.33 | 28 | 40mg gastro-resistant tablets |
| Paracetamol | 0.03 | 100 | 500mg caplets |
| Pizotifen | 0.09 | 28 | 1.5mg tablets |
| Pramipexole |  | 30 | 88mcg tablets |
| Prednisolone | 0.05 | 28 | 5mg tablets |
| Pregabalin | 0.07 | 84 | 100mg tablets |
| Pregabalin | 1.15 | 56 | 25mg capsules (Lyrica) |
| Prochlorperazine | 0.12 | 28 | 5mg tablets |
| Progesterone | 0.22 | 30 | 100mg capsules (Utrogestan) |
| Promethazine hydrochloride | 0.52 | 56 | 25mg tablets |
| Propranolol hydrochloride | 0.03 | 28 | 10mg tablets (Propranol) |
| Quinine | 0.46 | 28 | 200 mg (Quinine sulfate) |
| Ramipril | 0.04 | 28 | 2.5mg |
| Rivaroxaban | 1.80 | 28 | 20mg tablets (Xarelto) |
| Salbutamol | 0.01 | 200 | 100mcg/dose inhaler (Ventolin) |
| Sertraline | 0.04 | 28 | 50mg tablets |
| Sitagliptin | 1.19 | 28 | 25mg tablets (Januvia) |
| Sodium alginate with calcium carbonate and sodium bicarbonate | 0.00 | 500 | Calcium carbonate 16 mg per 1 ml, Sodium alginate 50 mg per 1 ml, Sodium bicarbonate 26.7 mg per 1 ml (Peptac liquid) |
| Sodium alginate with calcium carbonate and sodium bicarbonate | 0.02 | 150 | Calcium carbonate 16 mg per 1 ml, Sodium alginate 50 mg per 1 ml, Sodium bicarbonate 26.7 mg per 1 ml (Gaviscon) |
| Sodium chloride | 0.09 | 100 | 600mg (Slow Sodium) |
| Solifenacin succinate | 0.06 | 30 | 5mg tablets (Solifenacin) |
| Sumatriptan | 0.23 | 6 | 100mg tablets |
| Tapentadol |  | 28 | 50mg tablets (Palexia) |
| Testosterone | 0.57 | 50 | 20mg per 1 gram (Tostran 2% gel) |
| Testosterone | 1.04 | 30 | 40.5mg/2.5g gel sachets (Testogel) |
| Testosterone | 1.02 | 30 | 50mg/5g gel (Testim) |
| Tiotropium | 0.38 | 60 | 2.5mcg/dose inhalation solution (Spiriva Respimat) |
| Tobramycin | 13.93 | 56 | 300mg/5ml nebuliser solution (Nebuliser liquid) |
| Topiramate | 0.16 | 60 | 25mg tablets |
| Tramadol hydrochloride | 0.03 | 30 | 50mg capsules |
| Valsartan | 0.40 | 28 | 80mg tablets |
| Venlafaxine | 0.23 | 56 | 75mg tablets |
| Vitamins A and D | 0.09 | 28 | Vitamin A 4000 units, Vitamin D 400 units |
| Warfarin sodium | 0.06 | 28 | 3mg tablets (Warfarin) |
| Zopiclone | 0.07 | 28 | 3.75mg tablets (Sleeping tablets) |
| ^a^ Where appropriate, costs were inflated to 2022 prices using the NHS Hospital and Community Health Services Pay and Prices Inflation Index [1].  ^b^ References: NHS reference costs [2]; PSSRU 2022 [1]; British National Formulary [3].  **Abbreviation**: PSSRU: Personal Social Services Research Unit; LC: Long COVID; GP: General Practitioner | | | |

# Economic costs

| **Table A2** Regression coefficients for individual services received at LC specialist clinic regarding their impact on the longitudinal changes in health economic costs | | | | | | |
| --- | --- | --- | --- | --- | --- | --- |
| Model type: multiple linear regression^a,b^ | **Model (1): public sector cost [N=219]** | | **Model (2): wider cost [N=219]** | | **Model (3): total societal cost [N=219]** | |
|  | **Coeff. (£) (SE)** | ***P*-value** | **Coeff. (£) (SE)** | ***P*-value** | **Coeff. (£) (SE)** | ***P*-value** |
| Phone consultation | 52.2 (219.5) | 0.812 | 75.4 (231.7) | 0.745 | 179.8 (343.5) | 0.601 |
| Doctor consultation | **-522.1** (196.5)** | **0.009** | -43.0 (211.2) | 0.839 | -591.3 (310.3) | 0.058 |
| Physiotherapy | -454.5 (273.1) | 0.098 | -441.8 (288.7) | 0.128 | **-870.9* (426.1)** | **0.042** |
| Occupational therapy | -88.8 (272.7) | 0.745 | -119.9 (287.9) | 0.678 | -260.9 (426.7) | 0.542 |
| Speech & language therapy | **-1,972.4** (483.6)** | **<0.001** | -328.2 (532.0) | 0.538 | **-2,219.7** (773.2)** | **0.005** |
| Fatigue management | -183.5 (278.5) | 0.511 | -459.9 (292.5) | 0.118 | -698.2 (433.7) | 0.109 |
| Counselling | -65.1 (351.9) | 0.853 | 108.4 (371.5) | 0.771 | 15.3 (551.0) | 0.978 |
| Peer support group | -193.4 (595.0) | 0.745 | -891.6 (625.0) | 0.155 | -1,126.9 (928.3) | 0.226 |
| Dietitian | -64.9 (456.2) | 0.887 | -35.4 (481.7) | 0.942 | -203.6 (714.2) | 0.776 |
| Welfare advice | **-930.7* (464.8)** | **0.047** | -445.4 (494.9) | 0.369 | -1,381.0 (728.7) | 0.060 |
| Multidisciplinary team management | **-1,235.0** (454.6)** | **0.007** | 245.5 (488.9) | 0.616 | -949.3 (722.3) | 0.190 |
| Mental health | 414.2 (672.4) | 0.539 | -190.3 (710.5) | 0.789 | 268.0 (1,053.8) | 0.800 |
| Sleep management | **-3,550.0** (606.5)** | **<0.001** | -1,191.5 (690.4) | 0.086 | **-4,736.2** (973.0)** | **<0.001** |
| Imaging | -197.5 (659.8) | 0.765 | 80.8 (696.7) | 0.908 | -207.9 (1,033.3) | 0.841 |
| Other tests | -176.3 (438.9) | 0.688 | 332.4 (463.0) | 0.474 | 286.3 (687.3) | 0.677 |
| ^a^ Statistical significance: * *P*<0.05; ** *P*<0.01.  ^b^ All coefficients have been adjusted for duration between first and last response, age group, sex, ethnicity, IMD quintile, LC duration, region, hospitalisation for acute COVID-19 and change in EQ-5D-3L index.  **Abbreviation:** coeff.: coefficient; IMD: index of multiple deprivation; LC: Long COVID; N/A: not applicable; ref.: reference; SE: standard error | | | | | | |

# National economic impact

| **Table A3** Extrapolated national economic impact using the 2.5^th^ percentile of estimated longitudinal outcome trajectories | | | | | | | | | |
| --- | --- | --- | --- | --- | --- | --- | --- | --- | --- |
| Age group | Mean per-person impact from LC start to 7^th^ March 2024 | | | National prevalence [4] | National impact from LC start to 7^th^ March 2024 | | | National economic impact across outcomes | Annual national economic impact across outcomes^c^ |
|  | Monetary value of QALY loss^a^ | Public sector cost | Wider cost^b^ |  | Monetary value of QALY loss^a^ | Public sector cost | Wider cost^b^ |  |  |
| **Scenario:** constant impact from LC start to first observation; mean longitudinal trajectories between first and last observation maintained after last observation | | | | | | | | | |
| <35 years | £16,137 | £13,219 | £24,686 | 99,902 | £1,612,147,631 | £1,320,571,038 | £2,466,212,841 | £5,398,931,509 | £1,830,146,274 |
| 35-44 years | £19,351 | £6,757 | £26,073 | 61,638 | £1,192,731,061 | £416,480,179 | £1,607,096,018 | £3,216,307,259 | £1,061,487,544 |
| 45-54 years | £15,977 | £8,464 | £22,392 | 70,117 | £1,120,256,224 | £593,438,946 | £1,570,078,379 | £3,283,773,548 | £1,094,591,183 |
| 55-64 years | £12,831 | £13,359 | £21,963 | 69,917 | £897,093,101 | £934,019,968 | £1,535,565,210 | £3,366,678,279 | £1,089,539,896 |
| 65-74 years | £8,734 | £15,023 | £26,723 | 37,042 | £323,518,662 | £556,494,986 | £989,871,106 | £1,869,884,755 | £640,371,492 |
| ≥75 years | £3,617 | £8,977 | £16,869 | 21,605 | £78,151,953 | £193,952,989 | £364,450,147 | £636,555,089 | £223,352,663 |
| Total national impact across age groups | | | | | | | | £17,772,130,439 | £5,939,489,052 |
| **Scenario:** constant impact from LC start to first observation; mean longitudinal trajectories between first and last observation not maintained | | | | | | | | | |
| <35 years | £19,347 | £11,968 | £30,350 | 99,902 | £1,932,852,659 | £1,195,597,365 | £3,031,983,841 | £6,160,433,865 | £2,088,282,666 |
| 35-44 years | £20,722 | £6,425 | £33,455 | 61,638 | £1,277,250,021 | £396,030,375 | £2,062,113,282 | £3,735,393,679 | £1,232,803,194 |
| 45-54 years | £17,820 | £9,125 | £23,549 | 70,117 | £1,249,483,096 | £639,838,683 | £1,651,217,043 | £3,540,538,822 | £1,180,179,607 |
| 55-64 years | £15,631 | £13,088 | £24,296 | 69,917 | £1,092,886,725 | £915,107,070 | £1,698,733,729 | £3,706,727,524 | £1,199,588,196 |
| 65-74 years | £11,232 | £12,882 | £22,914 | 37,042 | £416,061,326 | £477,167,278 | £848,766,139 | £1,741,994,742 | £596,573,542 |
| ≥75 years | £6,191 | £7,639 | £15,360 | 21,605 | £133,755,762 | £165,030,009 | £331,856,855 | £630,642,625 | £221,278,114 |
| Total national impact across age groups | | | | | | | | £19,515,731,257 | £6,518,705,319 |
| ^a^ QALY loss monetised by using the cost-effectiveness threshold of £20,000 per QALY gained [5].  ^b^ Includes monetary values of productivity loss and informal care cost.  ^c^ According to EQ-5D-5L sample, the mean durations in years from LC start to 7^th^ March 2024 by age group were: 2.95 years for age <35 years; 3.03 years for age 35-44 years; 3.00 years for age 45-54 years; 3.09 years for age 55-64 years; 2.92 years for age 65-74 years; and 2.85 years for age ≥75 years. These were used to estimate the annual impacts.  **Abbreviation:** HEQ: health economics questionnaire; LC: Long COVID; QALY: quality-adjusted life year | | | | | | | | | |

| **Table A4** Extrapolated national economic impact using the 97.5^th^ percentile of estimated longitudinal outcome trajectories | | | | | | | | | |
| --- | --- | --- | --- | --- | --- | --- | --- | --- | --- |
| Age group | Mean per-person impact from LC start to 7^th^ March 2024 | | | National prevalence [4] | National impact from LC start to 7^th^ March 2024 | | | National economic impact across outcomes | Annual national economic impact across outcomes^c^ |
|  | Monetary value of QALY loss^a^ | Public sector cost | Wider cost^b^ |  | Monetary value of QALY loss^a^ | Public sector cost | Wider cost^b^ |  |  |
| **Scenario:** constant impact from LC start to first observation; mean longitudinal trajectories between first and last observation maintained after last observation | | | | | | | | | |
| <35 years | £21,103 | £22,703 | £57,298 | 99,902 | £2,108,222,591 | £2,268,120,837 | £5,724,228,427 | £10,100,571,854 | £3,423,922,662 |
| 35-44 years | £23,145 | £23,101 | £70,217 | 61,638 | £1,426,622,116 | £1,423,928,961 | £4,328,026,805 | £7,178,577,882 | £2,369,167,618 |
| 45-54 years | £19,362 | £12,969 | £47,559 | 70,117 | £1,357,601,118 | £909,326,811 | £3,334,716,253 | £5,601,644,182 | £1,867,214,727 |
| 55-64 years | £17,032 | £33,075 | £56,751 | 69,917 | £1,190,818,650 | £2,312,500,853 | £3,967,872,543 | £7,471,192,045 | £2,417,861,503 |
| 65-74 years | £17,394 | £19,181 | £93,307 | 37,042 | £644,326,035 | £710,516,363 | £3,456,290,745 | £4,811,133,144 | £1,647,648,337 |
| ≥75 years | £14,616 | £25,204 | £45,604 | 21,605 | £315,774,119 | £544,538,515 | £985,270,104 | £1,845,582,738 | £647,572,891 |
| Total national impact across age groups | | | | | | | | £37,008,701,845 | £12,373,387,738 |
| **Scenario:** constant impact from LC start to first observation; mean longitudinal trajectories between first and last observation not maintained | | | | | | | | | |
| <35 years | £21,609 | £22,373 | £40,739 | 99,902 | £2,158,778,979 | £2,235,130,490 | £4,069,937,848 | £8,463,847,317 | £2,869,100,785 |
| 35-44 years | £22,484 | £15,766 | £48,058 | 61,638 | £1,385,892,924 | £971,783,968 | £2,962,205,168 | £5,319,882,060 | £1,755,736,653 |
| 45-54 years | £19,403 | £13,589 | £31,748 | 70,117 | £1,360,514,018 | £952,817,833 | £2,226,049,952 | £4,539,381,803 | £1,513,127,268 |
| 55-64 years | £17,542 | £25,836 | £35,718 | 69,917 | £1,226,467,349 | £1,806,391,670 | £2,497,269,607 | £5,530,128,625 | £1,789,685,639 |
| 65-74 years | £15,598 | £22,463 | £61,737 | 37,042 | £577,789,032 | £832,058,530 | £2,286,849,619 | £3,696,697,181 | £1,265,992,185 |
| ≥75 years | £11,018 | £18,538 | £33,994 | 21,605 | £238,035,035 | £400,522,939 | £734,450,949 | £1,373,008,923 | £481,757,517 |
| Total national impact across age groups | | | | | | | | £28,922,945,909 | £9,675,400,048 |
| ^a^ QALY loss monetised by using the cost-effectiveness threshold of £20,000 per QALY gained [5].  ^b^ Includes monetary values of productivity loss and informal care cost.  ^c^ According to EQ-5D-5L sample, the mean durations in years from LC start to 7^th^ March 2024 by age group were: 2.95 years for age <35 years; 3.03 years for age 35-44 years; 3.00 years for age 45-54 years; 3.09 years for age 55-64 years; 2.92 years for age 65-74 years; and 2.85 years for age ≥75 years. These were used to estimate the annual impacts.  **Abbreviation:** HEQ: health economics questionnaire; LC: Long COVID; QALY: quality-adjusted life year | | | | | | | | | |

# References

1. Jones K, Weatherly H, Birch S, Castelli A, Chalkley M, Dargan A, et al. Unit Costs of Health and Social Care 2022. Personal Social Services Research Unit, Centre for Health Economics, editors. <https://www.pssru.ac.uk/project-pages/unit-costs/2022>.

2. NHS England. 2021/22 National Cost Collection data 2023. Available from: <https://www.england.nhs.uk/costing-in-the-nhs/national-cost-collection/>.

3. National Institute for Health and Care Excellence. British National Formulary (BNF) 2024 [updated 26th March 2024]. Available from: <https://bnf.nice.org.uk/>.

4. Office for National Statistics. Self-reported coronavirus (COVID-19) infections and associated symptoms, England and Scotland 2024 [cited 2024 2nd May]. Available from: <https://www.ons.gov.uk/peoplepopulationandcommunity/healthandsocialcare/conditionsanddiseases/datasets/selfreportedcoronaviruscovid19infectionsandassociatedsymptomsenglandandscotland>.

5. NICE. NICE health technology evaluations: the manual. Process and methods (PMG36). London: National Institute for Health and Care Excellence2022.
